# Sperm Originated Chromatin Imprints and LincRNAs in Organismal Development and Cancer

**DOI:** 10.1016/j.isci.2020.101165

**Published:** 2020-05-15

**Authors:** Santhilal Subhash, Meena Kanduri, Chandrasekhar Kanduri

**Affiliations:** 1 Department of Medical Biochemistry and Cell Biology, Institute of Biomedicine, Sahlgrenska Academy, University of Gothenburg, Gothenburg 40530, Sweden; 2 Department of Clinical Chemistry and Transfusion Medicine, Institute of Biomedicine, Sahlgrenska University Hospital 413 45, Gothenburg, Sweden

**Keywords:** Chromosome Organization, Endocrinology, Transcriptomics

## Abstract

Importance of sperm-derived transcripts and chromatin imprints in organismal development is poorly investigated. Here using an integrative approach, we show that human sperm transcripts are equally important as oocyte. Sperm-specific and sperm-oocyte common transcripts carry distinct chromatin structures at their promoters correlating with corresponding transcript levels in sperm. Interestingly, sperm-specific H3K4me3 patterns at the lincRNA promoters are not maintained in the germ layers and somatic tissues. However, bivalent chromatin at the sperm-specific protein-coding gene promoters is maintained throughout the development. Sperm-specific transcripts reach their peak expression during zygotic genome activation, whereas sperm-oocyte common transcripts are present during early preimplantation development but decline at the onset of zygotic genome activation. Additionally, there is an inverse correlation between sperm-specific and sperm-oocyte lincRNAs throughout the development. Sperm-lincRNAs also show aberrant activation in tumors. Overall, our observations indicate that sperm transcripts carrying chromatin imprints may play an important role in human development and cancer.

## Highlights

• Sp-lincRNAs carry distinct chromatin structures correlating with transcript levels
• Sp-lincRNAs are active during ZGA in preimplantation developmental stages
• SpOc-lincRNAs are active in pre-ZGA and decline at the onset of ZGA
• Sp-lincRNAs are silent post implantation but show aberrant cancer-specific activation

## Graphical Abstract

**Figure:**
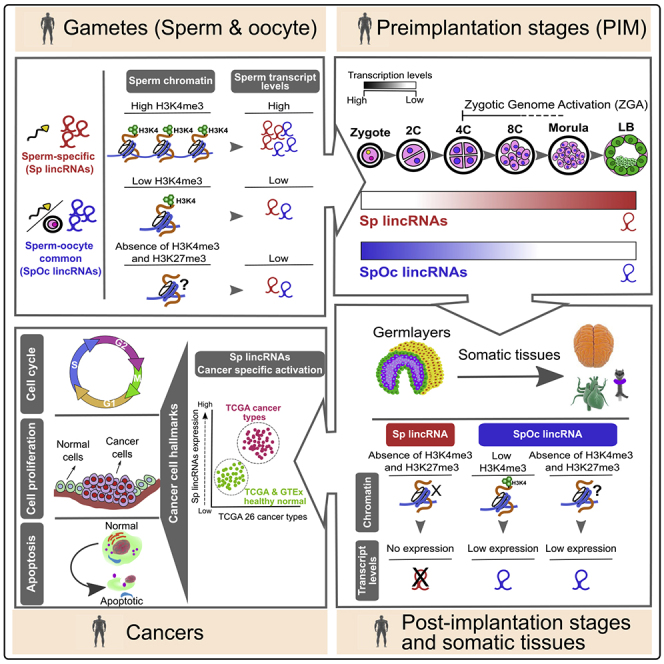

## Introduction

Maternal or oocyte-derived transcripts are known to regulate maternal to zygotic transition (MZT), which involves zygotic genome activation (ZGA) and maternal transcript degradation. These are the two important events necessary for subsequent preimplantation embryonic development (Xia et al., 2019). On the other hand, sperm with its compacted nucleus and minimal cytoplasm has long been considered transcriptionally incompetent and a passive vehicle that transmits merely paternal genome to the oocyte during fertilization. However, with the advent of high-throughput technologies, it is now clear not only that sperm contains RNA but also that its genome is highly structured and organized into distinct chromatin states dictated by positioned nucleosomes alongside protamine-enriched compact chromatin regions (Brykczynska et al., 2010, Hammoud et al., 2009, Paradowska et al., 2012). These observations suggest that not only oocyte but sperm also may take part in dictating genome organization and gene expression during MZT. Supporting this notion, overexpression of histone demethylation machinery (KDM1A/LSD1), which maintains H3K4 methylation levels, during spermatogenesis, had developmental defects that lasted for two subsequent generations (Siklenka et al., 2015). Thus, genetic and epigenetic information from both sperm and oocytes seem to have equal stakes in oocyte to embryo transition and also intergenerational transfer of acquired traits. Despite our increased understanding of sperm-dependent developmental regulation, how sperm-marked epigenetic states and sperm-derived transcripts contribute to gene expression at the embryonic and the adult stages of mammalian development remains unknown. Hence there is a need for an extensive investigation of transcriptionally competent chromatin regions across the sperm genome and this would immensely help in understanding their contribution to mammalian development. In this study, we attempt to integrate 17,705 human and mouse high-throughput sequencing samples comprising transcriptome (RNA sequencing [RNA-seq]) and histone chromatin profile datasets (H3K4me3 and H3K27me3 chromatin immunoprecipitation sequencing [ChIP-seq]) to explore the role of sperm-encoded information in development and disease regulation (Table S1). This study, in particular, emphasizes long noncoding RNAs (lncRNAs) as they have been shown as important lineage-specific developmental regulators, having cell-type- and stage-dependent functions (Cabili et al., 2011, Guttman et al., 2011, Zhang et al., 2014). Moreover, on lncRNAs, there exists only sporadic information regarding their significance in organism development. Therefore, there is a need for an integrative study to realize the importance of these transcripts, alongside protein-coding transcripts, through tracing their expression throughout the mammalian development.

Cancer testis antigens (CTAs) are a group of testis-specific proteins that show predominant expression on cancer cells. This observation suggests a functional connection between testis-specific expression and cancer. Majority of the CTA genes are preferentially expressed in testicular cell types such as spermatogonia or spermatocytes. Hence, we were particularly interested in characterizing whether sperm-derived RNAs show any preferential expression in different tumor types, which would in turn pave the way for understanding the contribution of sperm-derived transcripts in tumor development and progression (Simpson et al., 2005).

## Results

### Transcriptome-wide Identification of Sperm- and Oocyte-Associated Transcripts

**Figure 1:**
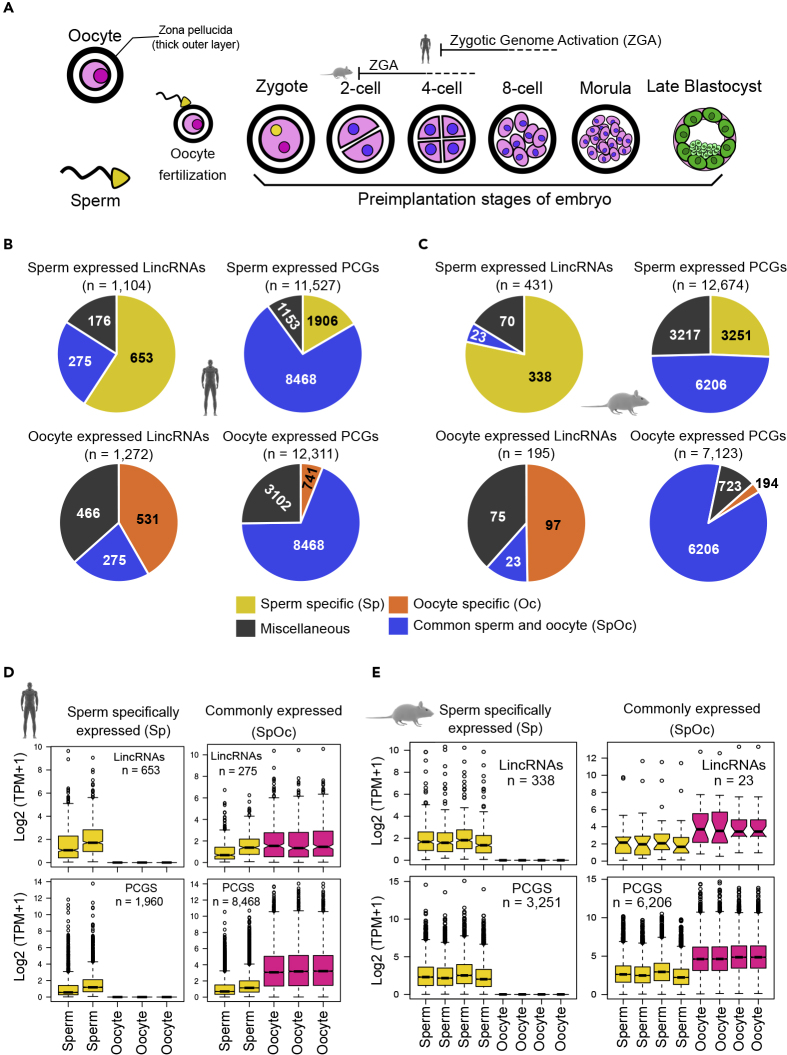
Comparative Transcriptome Analysis Reveals Similar Number of Transcripts Expressed in Sperm and Oocyte (A) A schematic of male and female germ cells and preimplantation embryo stages used for comparative transcriptomic analyses from both human and mouse. See also Table S1. (B and C) Venn diagrams showing the number of sperm and oocyte expressed long intergenic noncoding RNAs (lincRNAs) and protein-coding genes (PCGs) from human (B) and mouse (C) represented in three categories: sperm or oocyte (Oc) specific, commonly expressed in sperm and oocyte (SpOc), and Miscellaneous (inconsistently expressed between replicates samples from gametes). See also Table S2. (D and E) Boxplots from human (D) and mouse (E) showing the expression of sperm-specific and sperm-oocyte expressed (SpOc) lincRNAs and PCGs in sperm and oocyte. Box plots represent low expression range (lower whiskers), higher expression range, (upper whisker), median, inter quartile range (IQR), and the extreme expression values. See also Figure S1 and Table S3.

At the outset, we analyzed the transcriptome profiles of sperm, oocyte, and preimplantation embryo stages (two cell to late blastocyst) in human and mouse (Fan et al., 2015, Hammoud et al., 2014, Yan et al., 2013, Zhang et al., 2017) (Figure 1A). We included analysis on, in addition to protein-coding genes, long intergenic noncoding RNAs (lincRNAs) and other classes of noncoding transcripts (Other-ncRNAs), excluding lincRNAs, from the Ensembl transcript annotation (Figures S1A and S1D). For further interpretation of the data, we considered only intergenic transcripts or lincRNAs to avoid unnecessary noise from sequencing reads due to overlapping transcripts from the majority of non-stranded transcriptome datasets used in this study. This approach will increase the reliability and reproducibility of the results.

Our analysis show that sperm and oocyte, from both mouse and human, harbor comparable number of transcripts, and in particular, mouse harbors nearly twice as many transcripts in sperm compared with oocyte (Figures 1B and 1C). There were 653 lincRNA and 1,906 protein-coding gene (PCG) transcripts that were specifically present in human sperm, and hence we termed them as Sp-lincRNAs and Sp-PCGs, respectively. We found 531 lincRNAs and 741 PCGs to be human oocyte-specific (Oc-lincRNAs and Oc-PCGs), whereas 275 lincRNAs and 8,468 PCG transcripts were found to be expressed commonly in human sperm and oocyte (SpOc-lincRNAs and SpOc-PCGs). Transcripts that are inconsistently expressed between donors, within a gamete, were considered as miscellaneous and not considered for further analysis (Table S2) (Figure 1B). Like in humans, we categorized lincRNAs and PCGs from mouse sperm and oocyte expression data in a similar manner. In mouse, 338 lincRNA and 3,251 PCG transcripts were sperm specific (Sp-lincRNAs and Sp-PCGs); 97 lincRNAs and 194 PCG transcripts were oocyte specific (Oc-lincRNAs and Oc-PCGs). There were 23 lincRNA and 6,206 PCG transcripts commonly expressed between sperm and oocyte in mouse (SpOc-lincRNAs and SpOc-PCGs) (Table S2) (Figure 1C). Expression levels of SpOc-lincRNAs and SpOc-PCGs were higher in oocyte compared with sperm both in human and mouse (Figures 1D and 1E). Overall, there are a greater number of Sp-lincRNAs and Sp-PCGs compared with Oc-lincRNAs and Oc-PCGs in both mouse and human, which predicts a greater significance for the sperm-derived transcripts in mammalian development.

### Human Sperm Chromatin Is Well Structured and Correlates with Transcriptional Status

**Figure 2:**
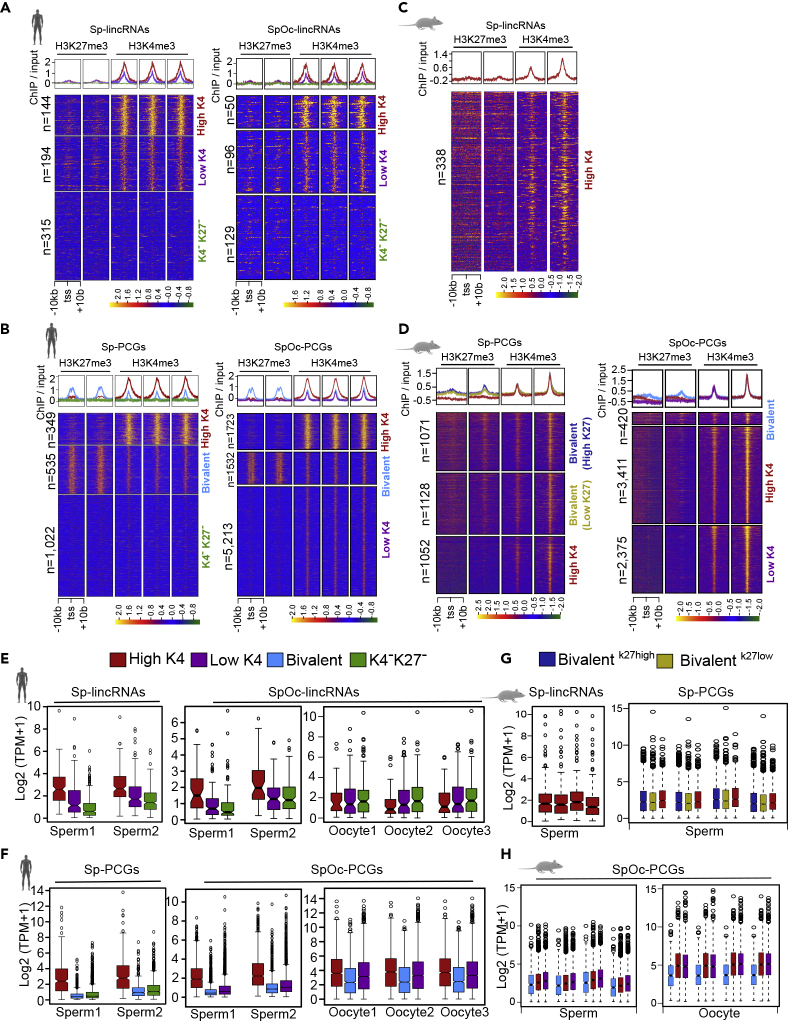
Sperm-Derived Chromatin Clusters Show Variable Expression Levels in Sperm (A and B) Based on H3K27me3 and H3K4me3 enrichment, human Sp and SpOc lincRNA (A) and PCG (B) promoters (extended ±10 kb from transcription start site, TSS) were categorized into three optimal sperm-derived chromatin clusters. See also Table S2. (C) Sp-lincRNA promoters (extended ±10 kb from transcription start site, TSS) from mouse show only high-K4 based on H3K27me3 and H3K4me3 enrichment. See also Table S2. (D) Sp and SpOc-PCG promoters (extended ±10 kb from transcription start site, TSS) from mouse show three sperm-derived chromatin clusters based on H3K27me3 and H3K4me3 enrichment. See also Table S2. (E and F) Expression status of Sp and SpOc-lincRNAs (E) and PCGs (F) from sperm-derived chromatin clusters in sperm and oocyte RNA-seq samples. (G and H) Expression status of Sp lincRNAs and PCGs (G) and SpOc PCGs (H) from sperm-derived chromatin clusters in sperm and oocyte samples. Heatmaps showing sperm-derived chromatin clusters were generated using k-means clustering. Box plots represent low expression range (lower whiskers), higher expression range (upper whisker), median, inter quartile range (IQR), and the extreme expression values. See also Figure S1 and Table S3.

Previously, it has been shown that paternal traits acquired in response to external stimuli can be inherited by offspring which, in particular, emphasizes a functional role for sperm chromatin structures in transgenerational inheritance (Gapp et al., 2018, Zhang et al., 2018). Consistent with the latter notion, based on ChIP-seq data, it was predicted that nearly 4% of the sperm genome is occupied by nucleosomes and that a significant portion of the retained nucleosomes were modified, specifically at noncoding RNA loci, imprinted genes, and developmental regulators (Hammoud et al., 2009, Jung et al., 2017). Considering these observations, we performed chromatin structure analyses at the promoter regions of Sp and SpOc transcripts to check whether they carry any distinct chromatin signatures and how they are maintained during the rest of the organismal development. Chromatin profiles of active (H3K4me3 enriched) and inactive (H3K27me3 enriched) histone marks at the Sp and SpOc gene promoters revealed several clusters based on the patterns of histone modifications (Hammoud et al., 2009, Jung et al., 2017) (Table S2). Analyses of human Sp-lincRNAs and SpOc-lincRNA promoters revealed three clusters: (1) high H3K4me3 (high-K4), (2) low H3K4me3 (low-K4), and (3) promoters lacking both H3K4me3 and H3K27me3 (K4^-^K27^-^) (Figure 2A). In Sp-PCG and SpOc-PCG promoters, we found chromatin clusters with high-K4, low-K4, bivalent chromatin (enriched with both H3K27me3 and H3K4me3 marks), and K4^-^K27^-^ (Figure 2B). In mouse, however, Sp-lincRNAs contain only a single cluster with high H3K4me3 at their promoters (Figure 2C). In the case of SpOc-lincRNAs, there were only a few candidates to be used for clustering (Figure 1C and Table S2). Mouse Sp-PCGs and SpOc-PCGs showed chromatin clusters having high-K4, low-K4, bivalent with high-K27, and bivalent with low-K27 marks (Figure 2D). We next compared the transcript levels of human and mouse Sp and SpOc transcripts with the chromatin modification patterns at their promoters. Interestingly, the transcript levels of human Sp (Sp-lincRNAs and Sp-PCGs) and SpOc (Sp-lincRNAs and SpOc-PCGs) transcripts correlated with the levels of H3K4me3 over their promoters (Figures 2A, 2B, 2E, and 2F). A higher transcript level was seen for the promoters with high-K4 compared with low-K4, bivalent, and K4^-^K27^-^ promoters. Interestingly, however, human SpOc-lincRNAs did not show cluster-specific transcript abundance in oocyte as seen in human sperm confirming that these chromatin profiles and transcript abundance patterns are sperm specific (Figures 2A and 2E). In the case of mouse, there was no correlation between chromatin patterns and the levels of sperm-specific (Sp-lincRNAs and Sp-PCGs) and commonly expressed transcripts (SpOc-lincRNAs and SpOc-PCGs) (Figures 2C, 2D, 2G, and 2H) (Tables S2 and S3). These observations indicate that there is a correlation between H3K4me3 enrichment at the promoters and the levels of their encoded transcripts in the human sperm, but such correlation was not evident in the mouse sperm. Similar analysis was performed on other classes of noncoding RNAs (Other-ncRNAs) by excluding lincRNAs and PCGs, and we did not find any correlation between chromatin patterns at their promoters and the encoded transcripts (Figures S1B–S1D). Thus, for further analysis, we have considered only lincRNAs to avoid unnecessary noise in the data due to ambiguously assigned reads to the overlapping transcripts from the majority of non-stranded transcriptome datasets used in this study.

### Transcripts from Sperm-Derived Chromatin Clusters Have Distinct Biological Functions in Human

**Figure 3:**
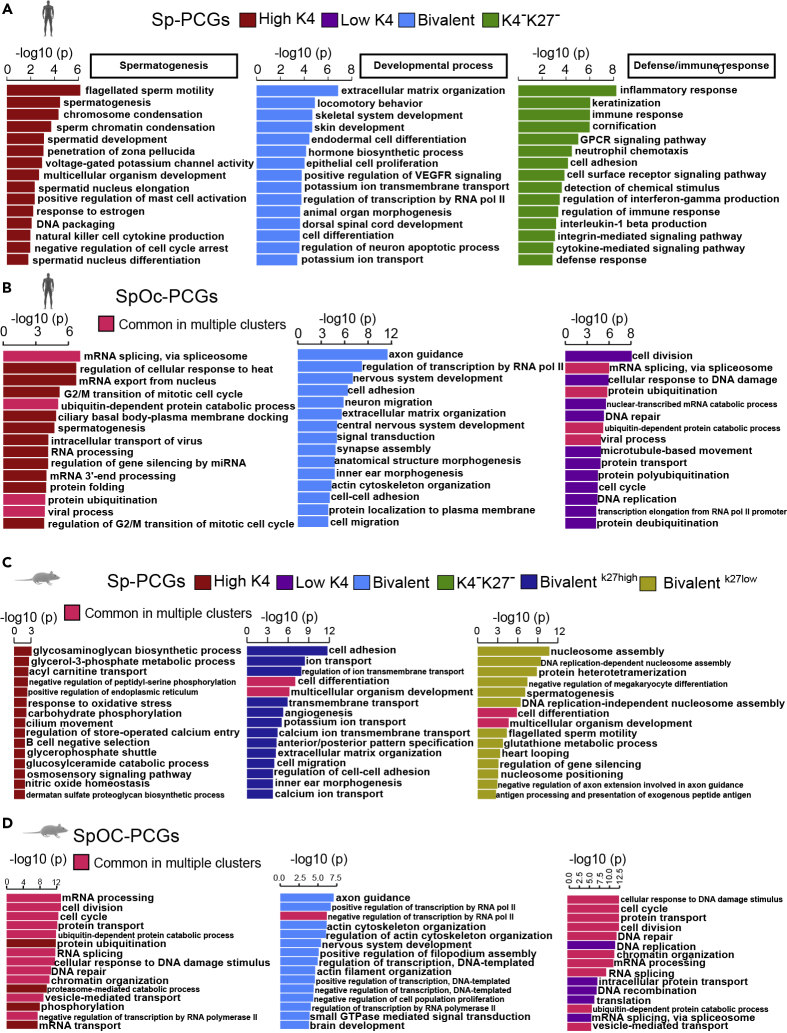
Sperm-Derived Chromatin Clusters Show Distinct Functional Profiles (A and B) Sperm-derived chromatin clusters of Sp-PCGs (A) and SpOc-PCGs (B) from human with enriched biological functions derived from gene ontology terms ranked using GeneSCF. (C and D) Sperm-derived chromatin clusters of Sp-PCGs (C) and SpOc-PCGs (D) from mouse with enriched biological functions derived from gene ontology terms ranked using GeneSCF. All gene ontology terms from the bar graphs were selected with enrichment p value < 0.05.

Intriguingly, PCGs from individual sperm-derived chromatin clusters fall into distinct biological functions in human. PCG cluster with high-K4 levels showed functions related to spermatogenesis such as flagellated sperm motility, sperm chromatin condensation, DNA packaging, and spermatid nucleus differentiation. Considering the enrichment of spermatogenesis-related functions in high-K4 group PCGs, we expect that lincRNAs from high-K4 clusters may play an important role in spermatogenesis regulation (Figure 3A). The cluster of Sp-PCGs having bivalent marks was predicted to be involved in biological functions such as organismal development, extracellular matrix organization, skeletal muscle development, VEGFR signaling, animal organ morphogenesis, and dorsal spinal cord development (Figure 3A). This observation corroborates the previous suggestion that bivalent domains are functionally linked to lineage commitment (Maezawa et al., 2018, Voigt et al., 2013). PCGs cluster devoid of histone marks (K4^-^K27^-^) showed immune defense-related functions such as immune response, neutrophil chemotaxis, and defense response (Figure 3A). SpOc-PCGs with high-K4 and low-K4 did not show distinct functions (Figure 3B).

Mouse PCG promoters having bivalent chromatin were also enriched in developmental processes as seen in humans, whereas the other chromatin clusters did not reveal any distinct functional profiles (Figures 3C and 3D). Additionally, this bivalent chromatin cluster, unlike in humans, were also enriched with spermatogenesis-related functions. These observations collectively suggest that the bivalent PCG groups from both mouse and human sperm may play an important role in the post-implantation stages of embryos and during the lineage commitments.

### Sperm Transcripts Show Temporal Expression during Preimplantation Development

**Figure 4:**
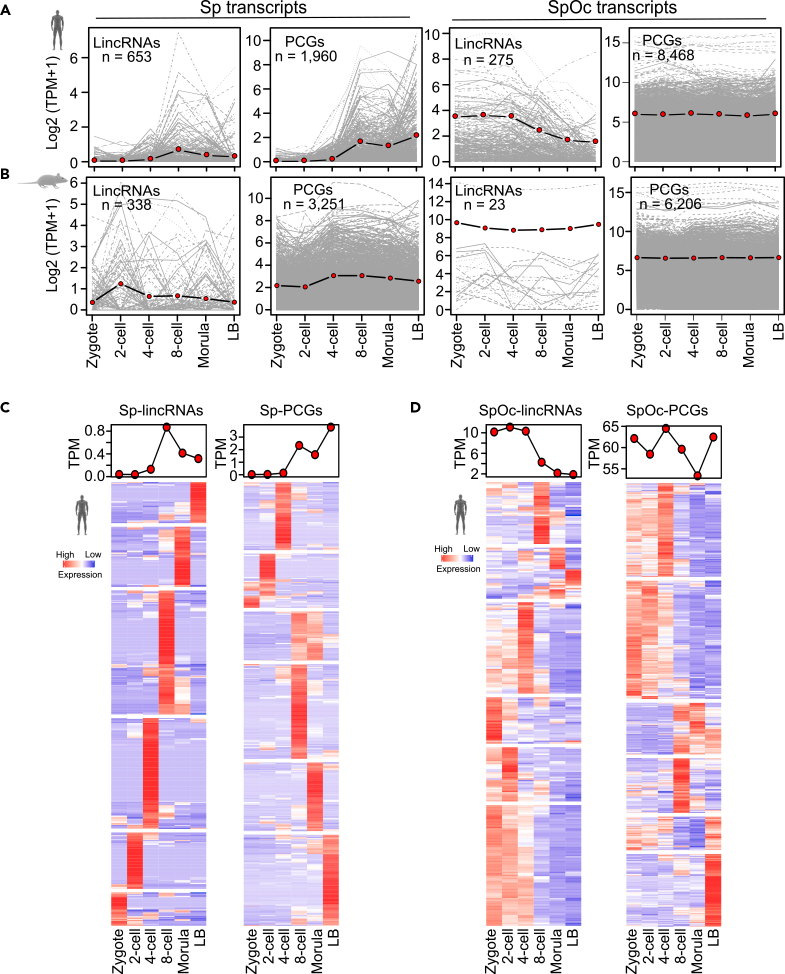
Sp and SpOc Show Temporal Expression during the Preimplantation Developmental Stages of Embryo (A and B) Expression of Sp- and SpOc transcripts (lincRNAs and PCGs) from human (A) and mouse (B) during the preimplantation stages of developing embryo (zygote, two-cell, four-cell, and eight-cell, morula, and late blastocyst). (C and D) Heatmaps showing stage-specific expression of Sp (C) and SpOc (D) transcripts (lincRNAs and PCGs) during preimplantation stage embryos. The expression values in plots (A and B) were log transformed to avoid extreme expression values. Expression profiles in heatmaps (C and D) were represented by *Z* score. See also Figure S2 and Table S3.

Since temporal and stage-specific gene expression patterns are strongly linked to function, we investigated whether Sp and SpOc transcripts possess temporal expression during preimplantation stages of development. We found that Sp-lincRNA and Sp-PCG transcripts were present at a very low level during early preimplantation stages but get activated between four- and eight-cell stages coinciding with ZGA (Figure 4A). SpOc transcripts showed similar expression patterns throughout the preimplantation development except SpOc lincRNAs, which showed gradual decrease in their levels from four cell stage coinciding with the commencing of ZGA (Figure 4B). Interestingly, Sp transcripts show exclusive stage-specific expression during preimplantation development between the four-cell stage and late blastocyst embryos, whereas SpOc transcripts show predominant expression between zygote and four-cell-stage embryos (Figures 4C and 4D). In particular, the expression of SpOc-lincRNAs, compared with SpOc-PCGs, is highly restricted to early preimplantation stages (Figures 4C and 4D). Exclusive stage-specific expression of Sp-lincRNAs suggests that they may play a critical role in preimplantation development.

We then investigated the temporal expression patterns of Sp and SpOc transcripts from the sperm-derived chromatin clusters during preimplantation development. Of note, Sp transcripts (Sp-lincRNAs and Sp-PCGs) from sperm-derived chromatin clusters show highly temporal expression patterns with no expression in early preimplantation-stage embryos and specific activation between four- and eight-cell stages of embryos (Figure S2A). Like in humans, in mouse, Sp-PCGs from sperm-derived chromatin clusters, which showed no expression during one- and two-cell early preimplantation embryos, become activated between two- and four-cell stages, coinciding with ZGA in mouse (Figure S2B).

Human SpOc transcripts from sperm-derived chromatin clusters showed a marginal decrease in expression between two- and four-cell stages. However, a marked decrease in expression was seen for transcripts from K4^-^K27^-^ and low-K4 sperm-derived chromatin clusters from four-cell stage onward. More importantly, SpOc PCGs from low-K4 group become super activated during preimplantation development (Figure S2A), whereas in mouse, Sp and SpOc transcripts from most of the sperm-derived chromatin clusters show opposing expression patterns across preimplantation stages (Figure S2B). Collectively, in mouse and human, sperm-specific transcripts from sperm-histone-derived clusters were present at very low level during early preimplantation embryos but get activated during ZGA, whereas SpOc transcripts, from sperm-histone-derived clusters (except the low-k4 cluster from mouse), remain active during early preimplantation development before getting inactivated during ZGA (Figures S2A and S2B). It is well known that maternal transcript and proteins (maternal detritus) are removed prior to the onset of ZGA (Hamm and Harrison, 2018). However, our data show that not just maternal (Oc) transcripts but also paternal transcripts were removed prior to ZGA, indicating involvement of both maternal and paternal or sperm transcript degradation (SpOc-TD) during early preimplantation development (Figures 4A–4D). Thus, our data open up investigations to explore further the sperm-derived transcripts in preimplantation development.

### Specific Loss of High-K4 and Low-K4 Chromatin Structures from the Sp-lincRNA Promoters in Post-implantation Development

**Figure 5:**
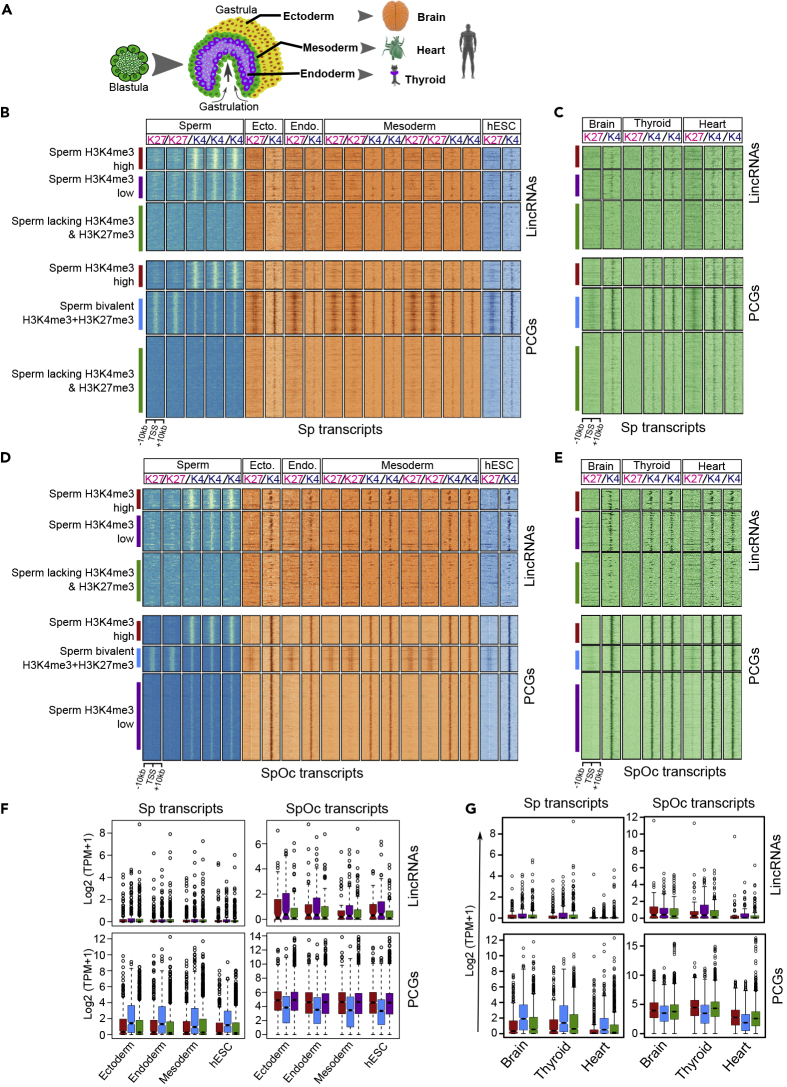
Sperm-Specific lincRNAs Are Not Critical for the Three Germ Layers Differentiation and Mature Tissue Formation (A) Schematic of samples chosen from three germ layers (ectoderm, mesoderm, and endoderm) and matured tissues (brain, heart, and thyroid), derived from respective germ layers. (B and C) Enrichment of H3K27me3 (K27) and H3K4me3 (K4) ChIP-seq signals from the three germ layers, human embryonic stem cells (hESC) (B), and the germ-layer-derived tissues (brain, thyroid, and heart) (C) over the promoters of Sp-lincRNA and PCGs (extended ±10 kb from TSS) from sperm-derived chromatin clusters. (D and E) Enrichment of ChIP-seq H3K27me3 (K27) and H3K4me3 (K4) signals from the three germ layers, hESCs (D), and the germ-layer-derived tissues (brain, thyroid, and heart) (E) at the promoters of SpOc-lincRNAs and PCGs (extended ±10 kb from TSS). (F) Expression of Sp- and SpOc-lincRNAs and PCGs in the three germ layers and hESCs. (G) Expression of Sp- and SpOc-lincRNAs and PCGs in brain, thyroid, and heart tissues. See also Figure S3 and Table S3.

During the process of gastrulation, the blastula begins to differentiate into specialized cell types giving rise to three distinct germ layers, namely, ectoderm (outer layer), mesoderm (middle layer), and endoderm (innermost layer) (Figure 5A). Each individual germ layer consists of multi-potent lineage-specific stem cells that can differentiate into different tissues types (Figure 5A). Therefore, we extended our analysis by investigating chromatin structure and expression patterns of Sp and SpOc transcripts in three germ layers and their derived somatic tissues (brain from ectoderm, thyroid from endoderm, and heart from mesoderm) (Chu et al., 2016, Davis et al., 2018, Friedman et al., 2018, Locke et al., 2015, Loh et al., 2014, Rada-Iglesias et al., 2011, Yan et al., 2013). We looked into the chromatin and expression patterns of Sp and SpOc transcripts from individual sperm-derived chromatin clusters during the post-implantation stages of the embryo and mature tissues. Strikingly, sperm-derived high-K4 and low-K4 chromatin structures from Sp-lincRNA promoters were lost in all the three germ layers and also the germ-layer-derived somatic tissues. Interestingly, however, the bivalent domains from Sp-PCG promoters maintained their bivalency (H3K4me3+H3K27me3) at their promoters in all the three germ layers and somatic tissues (Figures 5B, 5C, and S3A). Like high-K4 Sp-lincRNA promoters, high-K4 Sp-PCG promoters also lost their sperm-derived H3K4me3 levels in the germ layers and somatic tissues. Sperm-derived K4^-^K27^-^ clusters of Sp-lincRNAs and Sp-PCG transcripts maintained their K4^-^K27^-^ chromatin structures in the germ layers, but in somatic tissues there was a slight increase in H3K4me3 levels in Sp-PCG promoters (Figures 5B, 5C, and S3A).

High-K4 and low-K4 sperm-derived chromatin clusters from SpOc-lincRNAs showed low levels of H3K4me3 at their promoters, whereas K4^-^K27^-^ clusters maintain the same chromatin structure at their promoters in all the three germ layers and somatic tissues. Interestingly, sperm-derived chromatin clusters from SpOc-PCG promoters showed higher enrichment of H3K4me3 in all the three germ layers and their derived somatic tissues. However, sperm-derived bivalent domains from SpOc-PCG promoters maintained their bivalent chromatin structure in all the three germ layers but in the germ-layer-derived tissues only traces of bivalency were maintained (Figures 5D and 5E). Consistent with the lack of active histone marks, Sp-lincRNAs from all three sperm-derived chromatin clusters were not expressed in the three germ layers and somatic tissues (Figures 5F, 5G, and S3A). In contrast, Sp-PCGs having sperm-derived bivalent domain showed higher expression in germ layers as well as somatic tissues compared with the transcripts from high-K4 and K4^-^K27^-^ sperm-derived chromatin clusters (Figures 5F, 5G, and S3A). This observation is consistent with the potential of bivalent domains in lineage commitment (Maezawa et al., 2018). The sperm-derived high-K4 and low-K4 chromatin clusters of SpOc-lincRNAs have low levels of H3K4me3 with the corresponding low expression levels in all the three germ layers (Figures 5F, 5G, and S3A). All three sperm-derived chromatin clusters of SpOc-PCGs with high H3K4me3 levels exhibit higher expression (Figures 5F, 5G, and S3A).

Overall, these observations suggest that Sp-lincRNAs appear to be crucial for sperm maturation and preimplantation stage embryogenesis because of their highly cell-type- and developmental-stage-specific expression, whereas Sp-PCGs, SpOc-lincRNAs, and SpOc-PCGs seem to play crucial role in the formation of the three germ layers and somatic tissues. In particular, Sp-PCG transcripts with a bivalent chromatin at their promoters were present at low level in sperm owing to the higher levels of H3K27me3 in relation to H3K4me3 at their promoters, and their increased expression during the formation of germ layers and somatic tissues is correlated with an increase in H3K4me3 levels.

### Transcripts from Sperm-Derived High-K4 Chromatin Clusters Show Higher Expression in Round Spermatids

**Figure 6:**
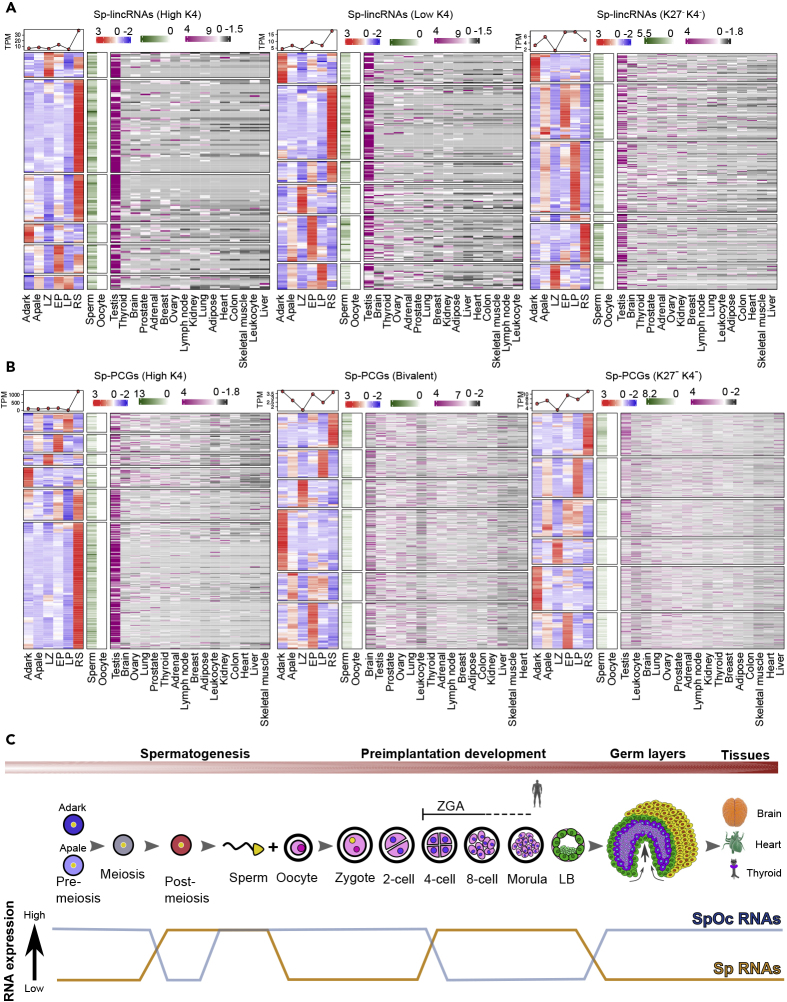
High-K4 Sp-lincRNAs and Sp-PCGS Are Abundantly Expressed in the Spermatid Stage of Spermatogenesis (A and B) Heatmaps showing the expression of Sp-lincRNAs (A) and PCGs (B) from individual sperm-derived chromatin clusters during different stages of spermatogenesis starting from pre-miosis (A-dark and A-pale spermatogonia), meiosis (leptotene/zygotene, early pachytene, and late pachytene), and post-meiosis (round spermatid). This heatmap is followed by status in sperm, oocyte, and 16 different tissues from human body map 2.0. (C) Model depicting observed expression patterns of sperm-specific and sperm-oocyte (SpOc) transcripts in gametes, preimplantation embryos, germ layers, and mature tissues. See also Figure S3.

The prevailing view has been that sperm, despite harboring active chromatin structures, lacks transcriptional activity. Therefore, we wanted to investigate whether these transcripts are derived directly from mature sperm or were already present during pre-meiotic, meiotic, and post-meiotic spermatogenic cell types and transmitted to sperm. For that, we used RNA-seq samples from pre-meiosis (A-dark and A-pale spermatogonia), meiosis (leptotene/zygotene, early pachytene, and late pachytene), and post-meiosis (round spermatid) stages of spermatogenesis (Jan et al., 2017). Sp-lincRNAs, Sp-PCGs, and SpOc-lincRNAs from high- and low-K4 sperm-derived chromatin clusters were expressed more in round spermatid during spermatogenesis and show more testis-specific expression (Figures 6A, 6B, S3B, and S3C), whereas Sp-lincRNA, Sp-PCG, SpOc-lincRNA, and SpOc-PCG transcripts from sperm-derived K4^-^K27^-^ and bivalent chromatin clusters were not expressed in round spermatids; rather, they showed cell-type-specific expression in other pre-meiotic and meiotic spermatogenic cell types with less specificity toward testis (Figures 6A, 6B, S3B, and S3C), indicating that these transcripts may be generated in mature sperm.

In summary, tracing the expression dynamics from different spermatogenic cell types, gametes (matured sperm and oocyte), and preimplantation (zygote to late blastocyst) and post-implantation stages (germ layers and matured tissues) reveal that Sp and SpOc transcripts have opposing expression patterns throughout the human development (Figure 6C).

### Transcription Factors Dictate the Expression of Sp- and SpOc-Transcripts in Preimplantation Development

**Figure 7:**
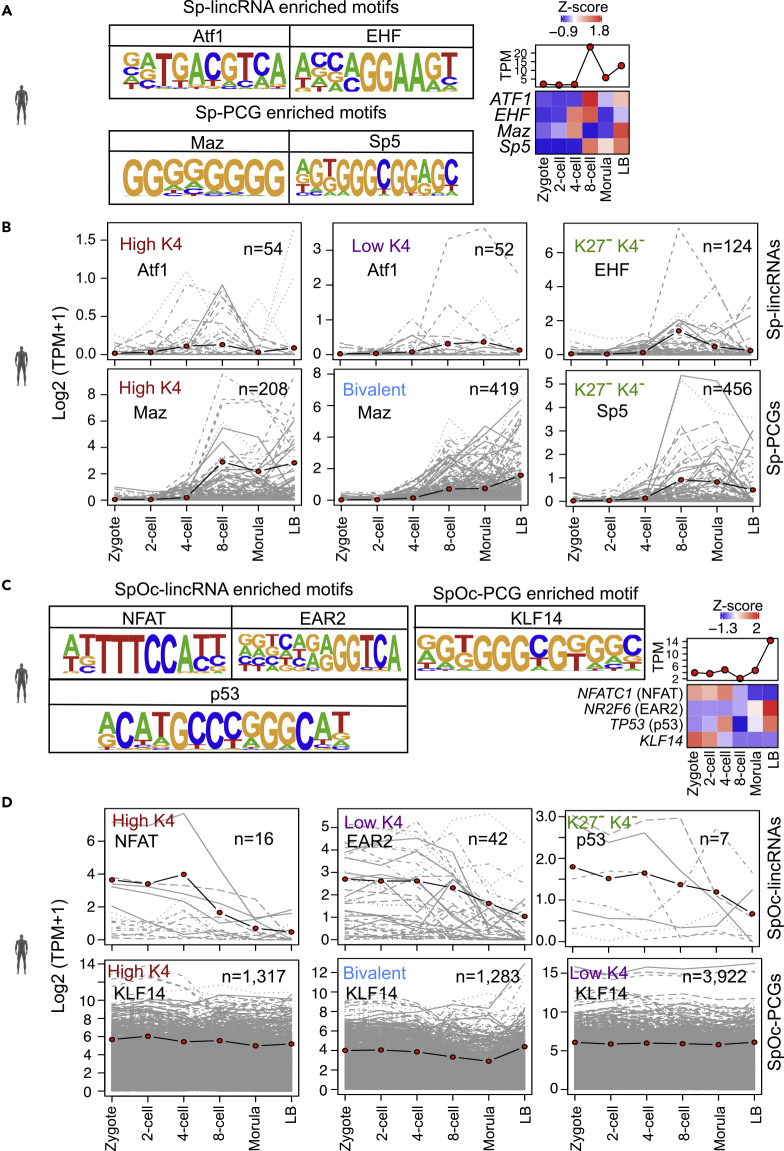
Preimplantation-Specific Transcription Factors Dictated the Expression of Sp-lincRNAs and Sp-PCGs during Maternal to Zygotic Transition (A) Transcription factor motifs enriched at the promoters of Sp-lincRNAs (Atf1 and EHF) and Sp-PCGs (Maz and Sp5). Heatmap showing the expression status of genes encoding these transcription factors in germ cells and preimplantation stages of embryo. (B) Expression of Sp transcripts, enriched with the indicated transcription factors, during preimplantation stages of embryo (two-, four-, and eight-cell, morula, and late blastocyst, LB). (C) Transcription factor motifs enriched at the promoters of SpOc-lincRNAs (NAFT, EAR2, and p53) and SpOc-PCGs (KLF14). Heatmap with the expression status of genes encoding these transcription factors in germ cells and preimplantation stages of embryo. (D) Expression of SpOc transcripts enriched with the indicated transcription factors during preimplantation stages of embryonic development (two-, four-, and eight-cell, morula, and late blastocyst, LB). See also Figures S4 and S5 and Table S3.

It is known that histone modifications along with *cis-* or *trans-*acting regulatory elements can control the transcription of genes (Luo et al., 2016). Transcription factors are known to play an important role in modulating gene expression either by activating or repressing the transcription. During early embryonic development there are many transcription factors involved in the transition of a zygote into fully developed embryo. Since Sp- and SpOc-transcripts are temporally expressed during preimplantation embryonic stages, we wanted to know what kind of transcription-factor-binding sites are enriched over these promoters. We used sequences from promoters (±250 bp from transcription start site, TSS) of these transcripts to find enriched motifs by matching with the known transcription factor motif sequences generated by HOMER using published ChIP-seq datasets. Among these factors, ATF1 (Activating Transcription Factor 1) and EHF (ETS Homologous Factor) were enriched significantly in the promoters of a greater number of Sp-lincRNA promoters (Figure S4A). Expression of genes encoding ATF1 and EHF transcription factors correlated with the expression of their Sp-lincRNA targets during ZGA (between four-cell and blastocyst) (Figures 7A and 7B). Similarly, Sp-PCG promoters were enriched with Maz and Sp5-binding sites, and also the expression of genes encoding these TFs corresponds to their target Sp-PCGs expression during ZGA (Figures S4B, 7A, and 7B). Sp-PCG transcripts from the sperm-derived K4^-^K27^-^ chromatin cluster of Sp-PCG transcripts have an Sp5 transcription factor motif, which is previously shown to be an important element in maintaining gene expression patterns during embryonic development (Treichel et al., 2001). We found NFAT (Nuclear Factor of Activated T Cells), EAR2 (ErbA-related protein 2), p53 (Phosphoprotein 53), and KLF14 (Kruppel Like Factor 14) transcription factors to be enriched in the promoters of SpOc-lincRNA and SpOc-PCG transcripts from sperm-derived chromatin clusters (Figures S5A, S5B, and 7C). Among these, only the expression of NFAT and KLF14 transcription factors was correlated with their target gene expression during early stages of preimplantation development (between zygote to four-cell) (Figures 7C and 7D). We did not see any correlation between the other transcription factors, EAR2 and p53, and their target gene expression during the preimplantation embryonic stages (Figures 7C and 7D). *KLF14* is known to have maternally derived expression, and it is necessary for embryonic and extra-embryonic tissue development (Parker-Katiraee et al., 2007). Also, NFAT deficiency is known to cause embryonic lethality in mice (Chuvpilo et al., 2002, Mak et al., 2011). These observations collectively suggest that the expression of most of the Sp and SpOc transcripts is driven by important transcription factors known to have a key role during embryonic development.

### Sp-lincRNAs Show Aberrant Expression in Cancer and Controls Cancer Cell Hallmarks

**Figure 8:**
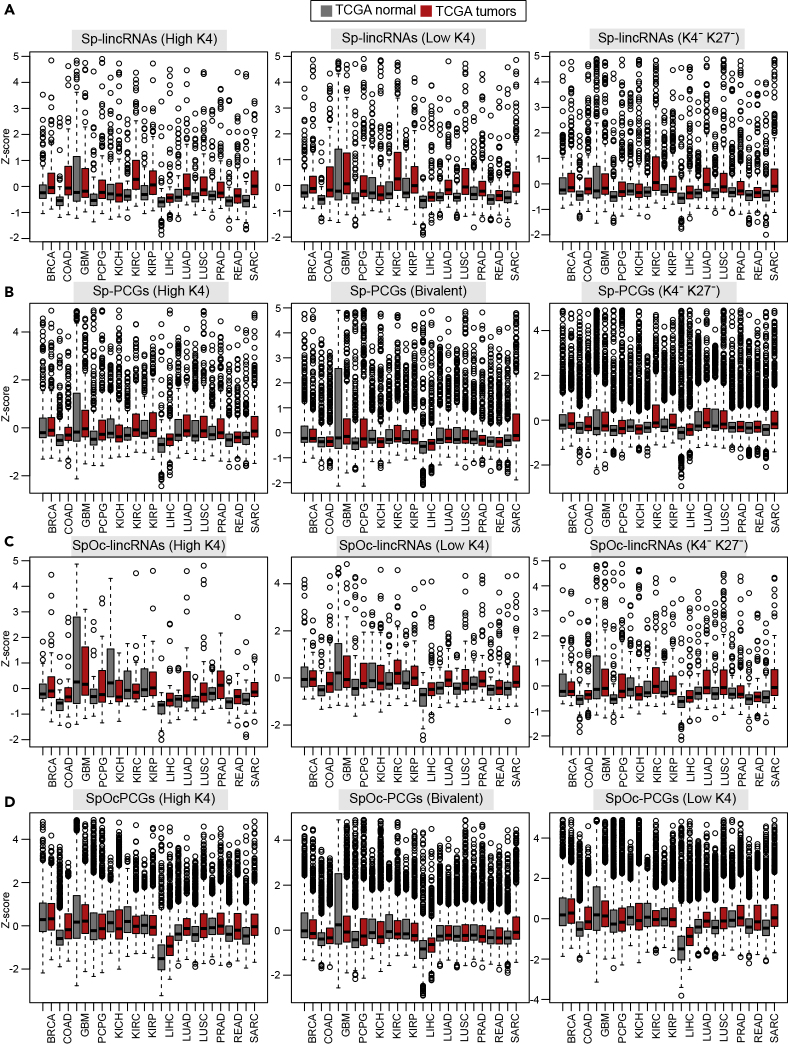
Sp-lincRNAs and Sp-PCGs Show Aberrant Expression in Different Cancers (A–D) Expression status of Sp-lincRNAs (A), Sp-PCGs (B), SpOc-lincRNAs (C), and SpOc-PCGs (D) in different tumors and corresponding healthy tissues from TCGA patient cohort. The *Z* score in the plot is derived from the normalized TPM expression values. See also Figures S6–S8 and Table S3.

**Figure 9:**
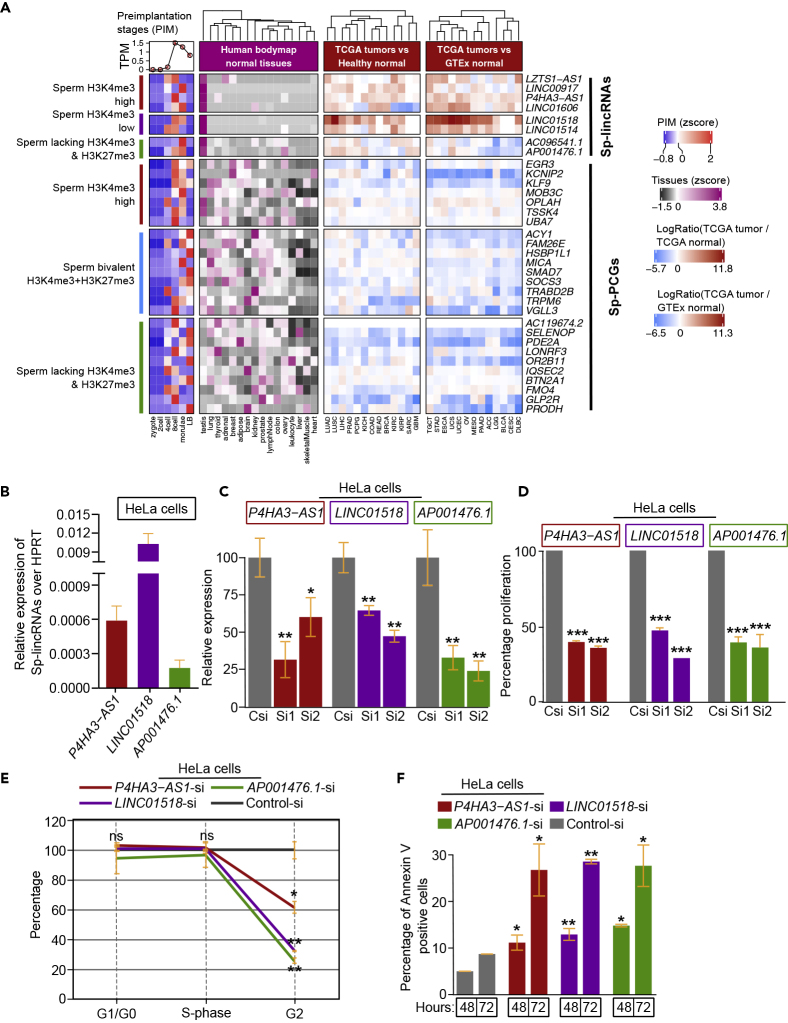
Sp and SpOc lincRNAs Show Tumor-Specific Activation (A) Status of Sp transcripts in germ cells, preimplantation stage embryos, the human body map tissues, TCGA tumors compared with the corresponding healthy samples, or TCGA tumors compared with GTEx health samples. *Z* score in the plots is derived from the normalized TPM expression values. The log fold change is calculated by comparing the expression of tumors with the health samples expression. (B) Relative expression of Sp transcripts in HeLa cell lines. (C) Percentage of relative expression levels of three Sp-lincRNAs that are downregulated using two different siRNAs and control siRNAs in HeLa cells. (D) MTT assay showing the percentage of proliferative cells for HeLa cells transfected with siRNAs for Sp-lincRNAs compared with the respective control siRNA samples. (E) Line graph showing the cell-cycle profiles of HeLa cells transfected with Sp-lincRNA siRNA and control siRNA. (F) Bar graphs representing the percentage of Annexin V-positive cells after 48 and 72 h transfection of HeLa cells with siRNA for three different Sp-lincRNAs and control siRNA samples. For each gene, the p value is calculated using two different siRNA-transfected samples. ∗ indicates p value < 0.05, ∗∗ indicates p value <0.01, and ∗∗∗ indicates p value <0.001. Data from plots are represented as mean ± SD. See also Figure S9 and Tables S3 and S4.

Previous studies have shown that RNAs involved in organism development and having testis-specific expression are known to take part in cancer development and progression (Aiello and Stanger, 2016, Hosono et al., 2017). To demonstrate the importance of Sp and SpOc transcripts in cancer, we used patient-derived RNA-seq samples from The Cancer Genome Atlas (TCGA). A comparison was made between TCGA tumors (N = 4,809) and corresponding TCGA healthy samples. Additionally, for a set of TCGA tumors (N = 4,035) for which there were no available TCGA normal samples, we used normal tissue samples from the GTEx consortium. Among the Sp and SpOc lincRNAs and PCGs, Sp-lincRNAs from sperm-derived chromatin cluster high-K4 showed more aberrant expression compared with the Sp-lincRNAs from low-K4 and K4^-^K27^-^ clusters (Figure 8A); thus, the level of their deregulation in cancers correlated with the extent of H3K4me3 enrichment at their promoters. However, Sp-PCGs from sperm-derived chromatin clusters did not reveal such correlation as seen with the Sp lincRNAs. SpOc lincRNAs and PCGs from sperm-derived chromatin clusters were less deregulated in cancers compared with the Sp-lincRNAs (Figure 8B). One common feature among the Sp and SpOc lincRNAs and PCGs is that transcripts from sperm-derived high-K4 clusters showed more deregulation than transcripts from lowK4, bivalent, and k4^-^K27^-^ chromatin clusters (Figures 8A–8D and S6–S8). A detailed look into the expression patterns of individual genes revealed some important previously known candidates from each chromatin cluster of Sp and SpOc transcripts (Figures 9A and S9A). Of note, we found an intergenic lncRNA *LINC01518* from Sp-lincRNAs group to be deregulated in 21 of the 26 cancers, showing high levels of deregulation in lung (LUAD and LUSC), prostate (PRAD), testis (TCGT), and uterine or ovary (UCS, UCEC, and OV)-related cancers.

Since Sp lincRNAs show higher expression in tumors compared with normal, we wanted to investigate whether they possess oncogenic properties. To this end, we investigated the expression of three Sp-lincRNAs (*P4HA3-AS1*, *LINC01518*, and *AP001476*.*1*), representing each of the three sperm-derived chromatin clusters, in human embryonic kidney cell line HEK-293 and cervical carcinoma cell line HeLa. We found that the Sp lincRNAs show higher expression in HeLa cells compared with the HEK293 cells (Figures 9B and S9B). We therefore next investigated the effect of their downregulation in HeLa cells, using siRNAs, on important cancer cell hallmarks such as cell proliferation, cell cycle progression, and apoptosis. Downregulation of the Sp lincRNAs significantly affected all the three cancer cell hallmarks, indicating that the three chosen Sp lincRNAs possess oncogenic properties (Figures 9C–9F and S9C).

These results suggest that Sp- and SpOc-lincRNAs, which are temporally expressed during preimplantation embryo development coinciding with ZGA and SpOc-TD, appear to play a crucial role in cancer development and progression.

## Discussion

Our study explores the potential role of genes that encode sperm-specific transcripts and possess sperm-inherited chromatin structures in mammalian development and cancer. Intriguingly, in humans, we found a comparable number of transcripts present in both sperm and oocyte, whereas in mouse, sperm has a greater number of transcripts compared with the oocyte. Our observations are consistent with previous data where a pool of 3,281 poly(A) transcripts were identified based on the microarray analysis of RNA from the ejaculated sperm of nine individuals (Ostermeier et al., 2002). The presence of significant number of sperm transcripts is interesting because sperm has been considered transcriptionally incompetent. This observation in particular gains importance considering that nearly 4% of the sperm genome retains histone-enriched chromatin, and more importantly, there are large islands of H3K4me3 at the promoter regions of sperm transcripts. The presence of RNA and active chromatin structures obviously points to potential of sperm chromatin supporting the transcriptional events. Consistent with the latter tenet, we observed correlation between the sperm transcript abundance and the extent of H3K4me3 enrichment at their promoters. Furthermore, previous investigations have shown sperm transcript alterations in response to motility, capacitation, and cryopreservation (Ren et al., 2017). However, this is a far-fetched conclusion considering that there is a lack of data supporting the active transcription machinery at the H3K4me3 enriched promoters in sperm. This raises an important question, from where do sperm transcripts originally originate? Previously, it was suggested that the transcript accumulation in sperm is not a stochastic event rather a preservation of RNA through an organized process throughout spermiogenesis (Krawetz, 2005). Consistent with the latter notion, our data show that promoters of transcripts from sperm-derived chromatin cluster with high H3K4me3 show higher expression in spermatid, compared with the other spermatogenic cell types. This observation may indicate that sperm could inherit these transcripts from spermatid. However, it is not clearly evident how the accumulation of transcripts in sperm from the other sperm-derived chromatin clusters (low-K4 and K4^-^K27^-^) occurs, even though their expression is restricted to meiotic or pre-meiotic spermatogenic cell types. These observations indicate that low levels of transcription may persist during sperm maturation, and this notion is consistent with the data that sperm transcripts differs in caput and cauda and moreover, as discussed earlier, sperm RNA alterations in response to motility and capacitation (Conine et al., 2018, Ren et al., 2017). Thus, future investigations along similar lines will be fruitful.

The next important question is whether genes encoding sperm-specific transcripts and/or the transcripts per se have any role in preimplantation development. Multiple lines of evidence suggest that sperm transcripts play an important role in early preimplantation development. For example, defective blastocyst embryos generated from dicer knockout germ cells can be rescued with sperm RNA (Yuan et al., 2016). Similarly, embryos developed from caput sperm had post-implantation developmental defects compared with cauda sperm, which give rise to embryos that develop to term. Interestingly, microinjection of cauda-specific small RNAs into caput-derived embryos rescued post-implantation embryonic lethality (Conine et al., 2018). Additionally, sperm RNA has been shown to play an important role in modulating the RNA levels during fertilization, first cleavage, and blastocyst of early stages of embryo development (Alves et al., 2019, Bohacek and Rassoulzadegan, 2020). In line with this, our gene ontology analysis of sperm transcripts from high-K4 chromatin cluster reveals biological functions related to spermatogenesis, penetration to zona pellucida, ion channel activity, etc. Sperm penetration to the zona pellucida and ion channel activity are the most important functions during fertilization, and it seems that sperm transcripts play an important role in these biological processes. These observations, collectively, not only emphasize the importance of sperm transcripts in early preimplantation development but also highlight the global transcriptome dynamics during sperm maturation. Thus, the identification of a significant number of sperm transcripts in our investigation signifies the importance of sperm transcripts in early preimplantation development. Interestingly, sperm-derived bivalent chromatin clusters were enriched with biological functions related to developmental processes (Tomizawa et al., 2018), which is consistent with previous studies on the association of genes harboring bivalent chromatin promoters with developmental functions. These kind of distinct clusters of biological processes dictated by sperm transcripts were not seen in sperm-oocyte-expressed transcripts. In mouse, on the other hand, there was no correlation between chromatin structure at the promoters and the levels of their encoded transcripts in sperm, and also, we did not find any distinct biological functions enriched for individual clusters as seen in the human sperm. These observations clearly indicate that the human sperm genome is highly structured to execute important developmental functions and that sperm-specific lincRNAs appear to have a greater biological role in sperm maturation and preimplantation development.

Another interesting aspect of the current investigation is the temporal expression of genes that encode sperm-specific and sperm-oocyte transcripts during preimplantation development. Genes that encode sperm-specific transcripts carry distinct chromatin structures and show stage-specific expression across preimplantation stages. This exclusive stage-specific expression is not seen with genes that encode sperm-oocyte transcripts, rather the majority of these transcripts show expression during early preimplantation stages, primarily between two- and four-cell stages and start to decline or degrade during the onset of ZGA. Highly temporal expression of genes that encode sperm-specific transcripts in the preimplantation stages signifies their importance during early preimplantation development. Moreover, genes that encode sperm-specific and sperm-oocyte transcripts have crucial non-overlapping roles in preimplantation development. It is reasonable to assume that the sperm-inherited chromatin imprints together with the help of preimplantation development associated transcription factors may bring in the observed stage-specific expression (Bui et al., 2011). Indeed, this seems to be the case as sperm transcripts harbor motifs for transcription factors that show specific expression during early embryonic development. Thus, it is more likely that an interplay between sperm-inherited chromatin imprints and early embryonic-specific transcription factors may in part contribute to the stage-specific expression of the genes that encode sperm and sperm-oocyte transcripts.

Strikingly, the expression of Sp-lincRNAs as well as their promoter epigenetic profiles were lost in the three germ layers and their derived somatic tissues. This again emphasizes the importance of the genes that encode sperm lincRNAs in fertilization and preimplantation development. On the other hand, genes that encode Sp-PCGs and SpOc transcripts (SpOc-lincRNAs and SpOc-PCGs) continue to express in all the three germ layers and the germ-layer-derived multiple tissues. Comparison of the histone profiles of sperm, germ layers, and mature tissues revealed that Sp-PCGs and SpOc-PCGs retain their sperm-specific bivalent chromatin in the germ layers and somatic tissues; however, the ratio of H3K4me3 and H3K27me3 is different from that in the sperm. These observations indicate that the sperm-derived bivalent chromatin imprints at the Sp-PCGs and SpOc-PCGs promoters are maintained during the germ layers formation and these sperm-inherited chromatin imprints may play an important role in crucial developmental decisions by regulating spatiotemporal expression of Sp-PCGs and SpOc-PCGs. Thus, our study found two sets of genes: one set, sperm-specific genes with high-K4 promoters, whose transcripts are enriched in sperm, activated during ZGA and transcriptionally silenced in the three germ layers and somatic tissues. In the other set of genes, comprising Sp-PCGs, SpOc-lincRNAs, and SpOc-PCGs with high-K4, low-K4, K4^-^K27^-^, and bivalent (H3K4me3/H3K27me3) promoters, transcripts are enriched in sperm, and they express throughout preimplantation development, the germ layers, and in multiple somatic tissues. Thus, sperm-encoded information seems to take part in important developmental decisions.

Previous studies have proposed a functional link between early embryonic gene expression program and cancer development and progression (Bouckenheimer et al., 2016, Shah et al., 2018). In particular, genes active during preimplantation development are deregulated in several cancers. Moreover, cancer/testis expression has long been serving as basis for identifying diagnostic and prognostic markers for several cancers. Our data on Sp-lincRNAs having high-K4 at the promoters in sperm are particularly striking as they show higher deregulation in multiple cancers compared with lincRNAs from the other sperm-derived chromatin clusters (bivalent or K4^-^K27^-^). Interestingly, the level of their deregulation in cancer matches with the sperm H3K4me3 levels at the promoters. For example, lincRNAs with high-K4 levels at their promoters showed more deregulation in cancer compared with the lincRNAs with low-K4, indicating that mechanisms that establish sperm-specific chromatin imprints are recapitulated in cancer. Their higher expression in tumors compared with the corresponding normal tissues indicate that they may behave as oncogenes. We have also tested the oncogenic properties of selected lncRNAs from high-K4, low-K4, and K4^-^K27^-^ clusters in HeLa cell line by measuring their effect on crucial cancer cell hallmarks that define the oncogenic drivers such as cell proliferation, cell cycle progression, and apoptosis. Loss of function of these lncRNAs in HeLa cells using siRNAs resulted in decrease in cell proliferation, cell cycle progression, and apoptosis, indicating that these sperm lincRNAs harbor oncogenic properties. Thus, Sp-lincRNAs may serve as comprehensive resource for diagnostic/prognostic markers and potential therapeutic targets. SpOc-lincRNAs also showed deregulation in multiple cancers but to a lesser extent compared with the Sp-lincRNAs. Surprisingly, Sp-PCGs and SpOc-PCGs were mostly downregulated in cancers, and this in particular is consistent with their role in immune response regulation as immune response genes are mostly suppressed in many cancers.

In sum, the abundance of RNA, in particular lincRNAs, in sperm necessitates further investigation on their importance in sperm maturation, fertilization, and preimplantation development and cancer. More importantly, our study laid a strong basis for further investigation on the functional role of sperm-inherited chromatin imprints in organismal development.

### Limitations of the Study

Lack of chromatin data (ChIP-seq) for different stages of human preimplantation embryos limited our possibility to explore whether sperm-derived chromatin imprints are preserved during preimplantation development. Exploring this would have added an in-depth resolution to our analysis.

### Resource Availability

#### Lead Contact

Correspondence to lead contact Chandrasekhar Kanduri, E-mail: kanduri.chandrasekhar@gu.se.

#### Materials Availability

This study did not generate any new sequencing data. Analysis was performed using public datasets.

#### Data and Code Availability

The processed datasets generated during this study are available publicly at Mendeley Data repository with the following DOI https://dx.doi.org/10.17632/695c4zvr6d.1.

## Methods

All methods can be found in the accompanying Transparent Methods supplemental file.

## Supplemental Information

Document S1. Transparent Methods, Figures S1–S9, and Tables S4

Table S1. Studies from Which the Samples Are Taken for the Analysis, Related to Figures 1–9 and S1–S9

Table S2. List of lincRNA and Protein Coding Gene (PCG) Candidates Obtained from the Analysis, Related to Figure 1

Table S3. List of all Individual Processed Files and Their Deposited Links, Related to Figures 1–9 and S1–S9
