# Supplementary material for: Sperm Originated Chromatin Imprints and LincRNAs in Organismal Development and Cancer
Source: iScience. 2020 May 15;23(6):101165. doi: 10.1016/j.isci.2020.101165 (PMC7262563; doi:10.1016/j.isci.2020.101165)
Supplement: Document S1. Transparent Methods, Figures S1–S9, and Tables S4 [file mmc1.pdf]

iScience, Volume *23*

Supplemental Information

Sperm Originated Chromatin Imprints and LincRNAs in Organismal Development and Cancer

Santhilal Subhash, Meena Kanduri, and Chandrasekhar Kanduri

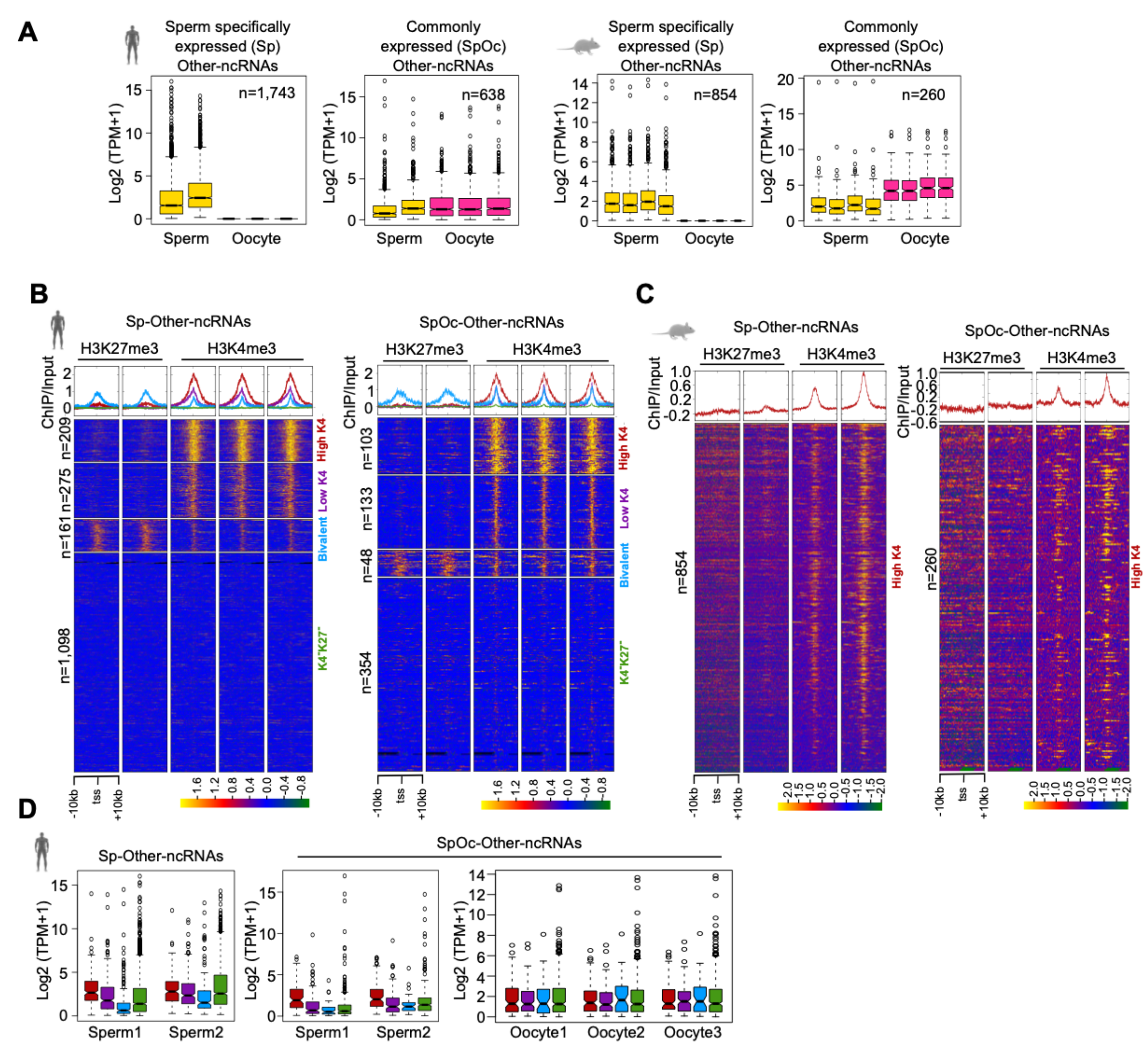

Figure S1. Transcriptome and chromatin profiles of Other-ncRNAs, Related to Figure 1. A) Boxplots from human (**left**) and mouse (**right**) showing the expression of sperm-specific and sperm-oocyte expressed (SpOc) lincRNAs and PCGs in sperm and oocyte. Box plots represent low expression range (lower whiskers), higher expression range (upper whisker), median, inter quartile range (IQR) and the extreme expression values. **B-C**) Based on H3K27me3 and H3K4me3 enrichment, Sp- and SpOc-Other-ncRNAs promoters (extended ± 10 kb from transcription start site, TSS) from human (**B**) and mouse (**C**) were categorized into three optimal sperm derived chromatin clusters. **D**) Expression status of human Sp and SpOc-Other-ncRNAs from sperm derived chromatin clusters in sperm and oocyte RNA-seq samples.

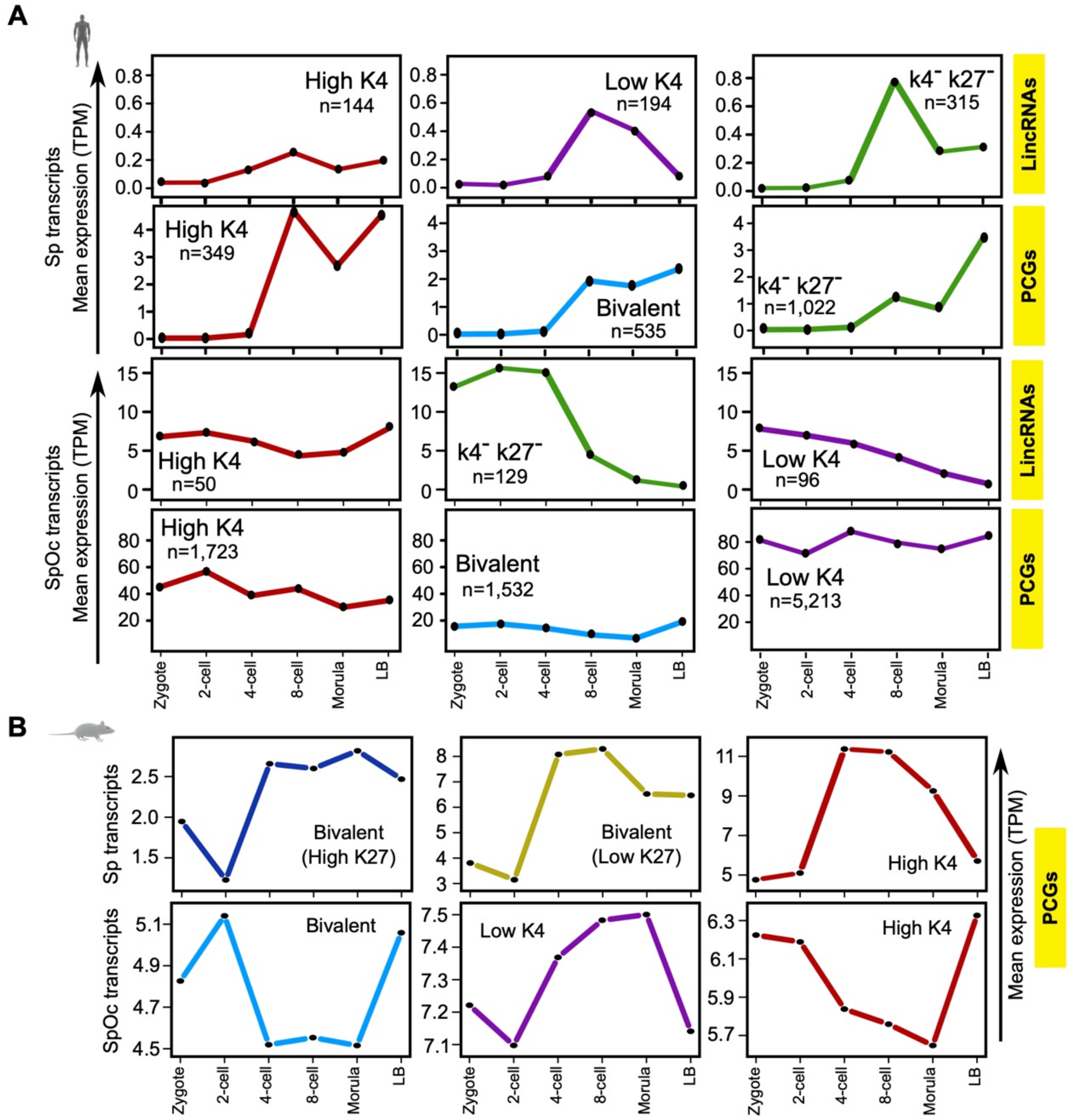

Figure S2. Sperm derived chromatin clusters show distinct stage-specific and non-overlapping gene expression during preimplantation development, Related to Figure 4. A-B) Sp- and SpOc transcripts (lincRNAs and PCGs) from sperm derived chromatin clusters showing cluster-wise expression during the preimplantation stages of developing embryo in human (**A**) and mouse (**B**). Mean expression is calculated from normalized TPM values.

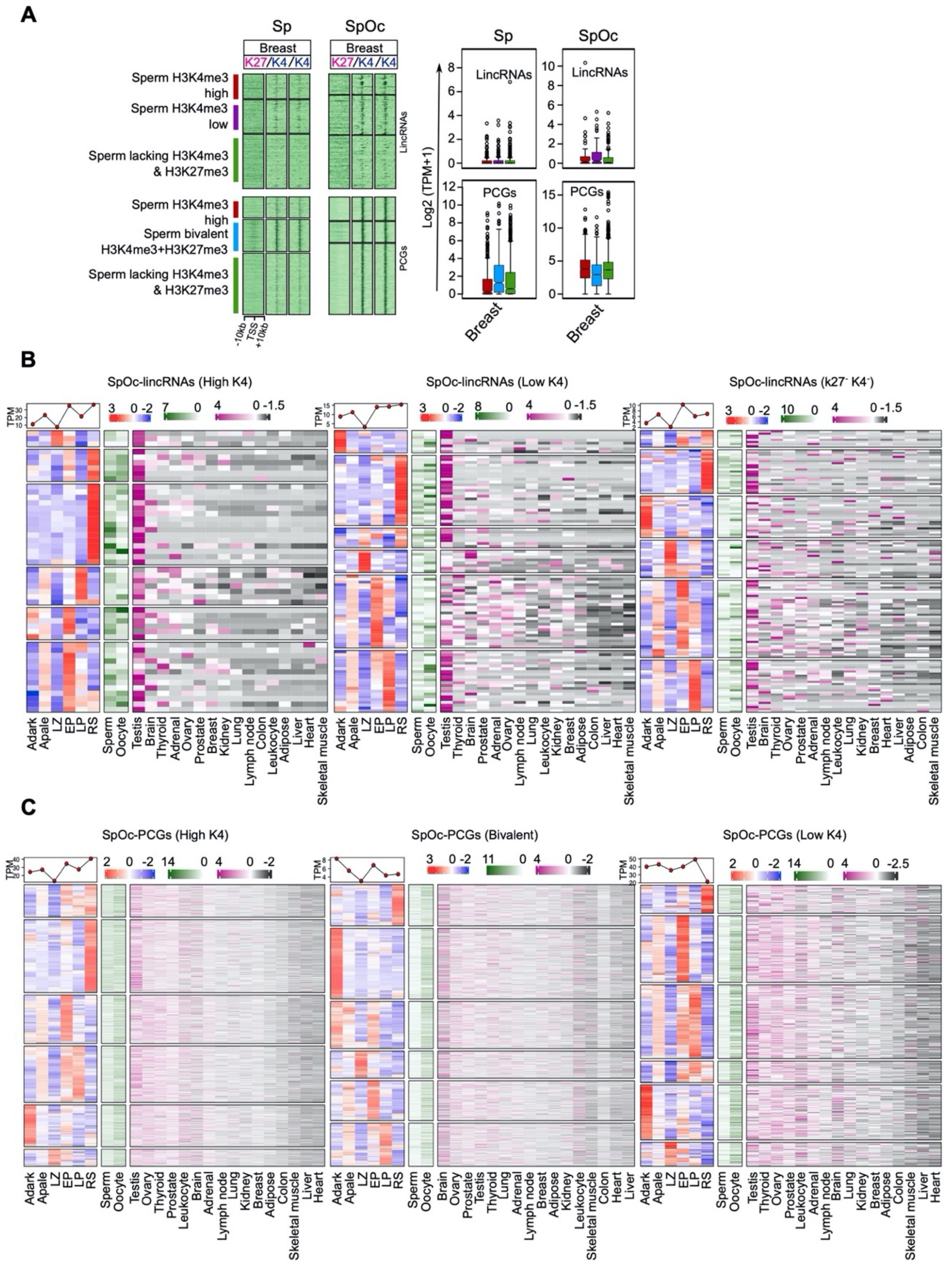

Figure S3. Sp and SpOc transcripts status in matured tissue and during spermatogenesis, Related to Figure 5 and Figure 6. A) Enrichment of H3K27me3 (K27) and H3K4me3 (K4) ChIP-seq signals over the promoters (± 10 kb) of Sp and SpOc lincRNAs and PCGs from sperm derived chromatin clusters in the ectoderm derived mature tissue (breast). Boxplots showing the expression of human Sp and SpOc transcripts (lincRNAs and PCGs) in normal breast tissue. **B-C**) Heatmaps showing the expression of SpOc-lincRNAs (**B**) and PCGs (**C**) from sperm derived chromatin clusters in spermatogenic cell types, sperm, oocyte and in 16 different tissues from the human body map 2.0.

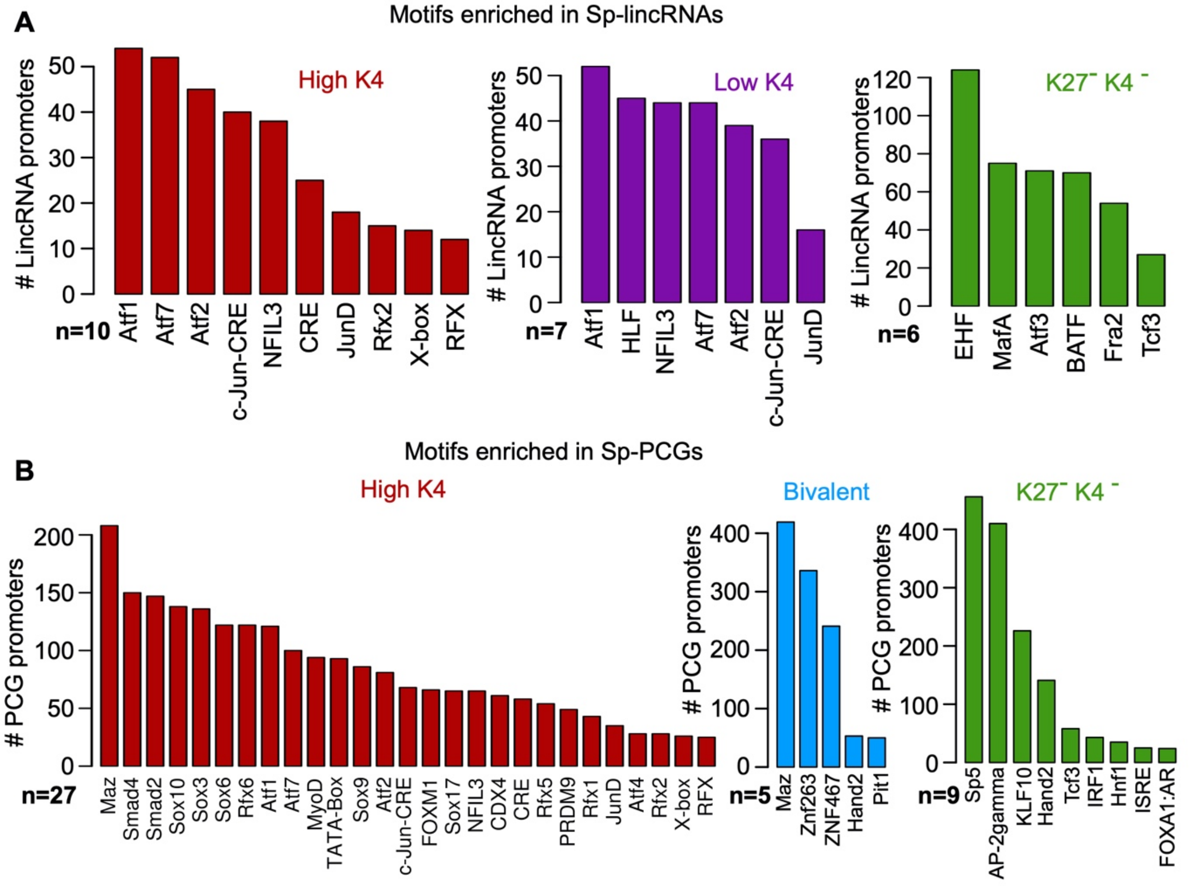

Figure S4. Frequency of transcription factor motifs in human Sp transcripts, Related to Figure 7. A-B) Bar graphs with the number of associated human Sp-lincRNA (**A**) and Sp-PCG (**B**) promoters from three sperm derived chromatin clusters of Sp transcripts enriched with the indicated transcription factor motifs.

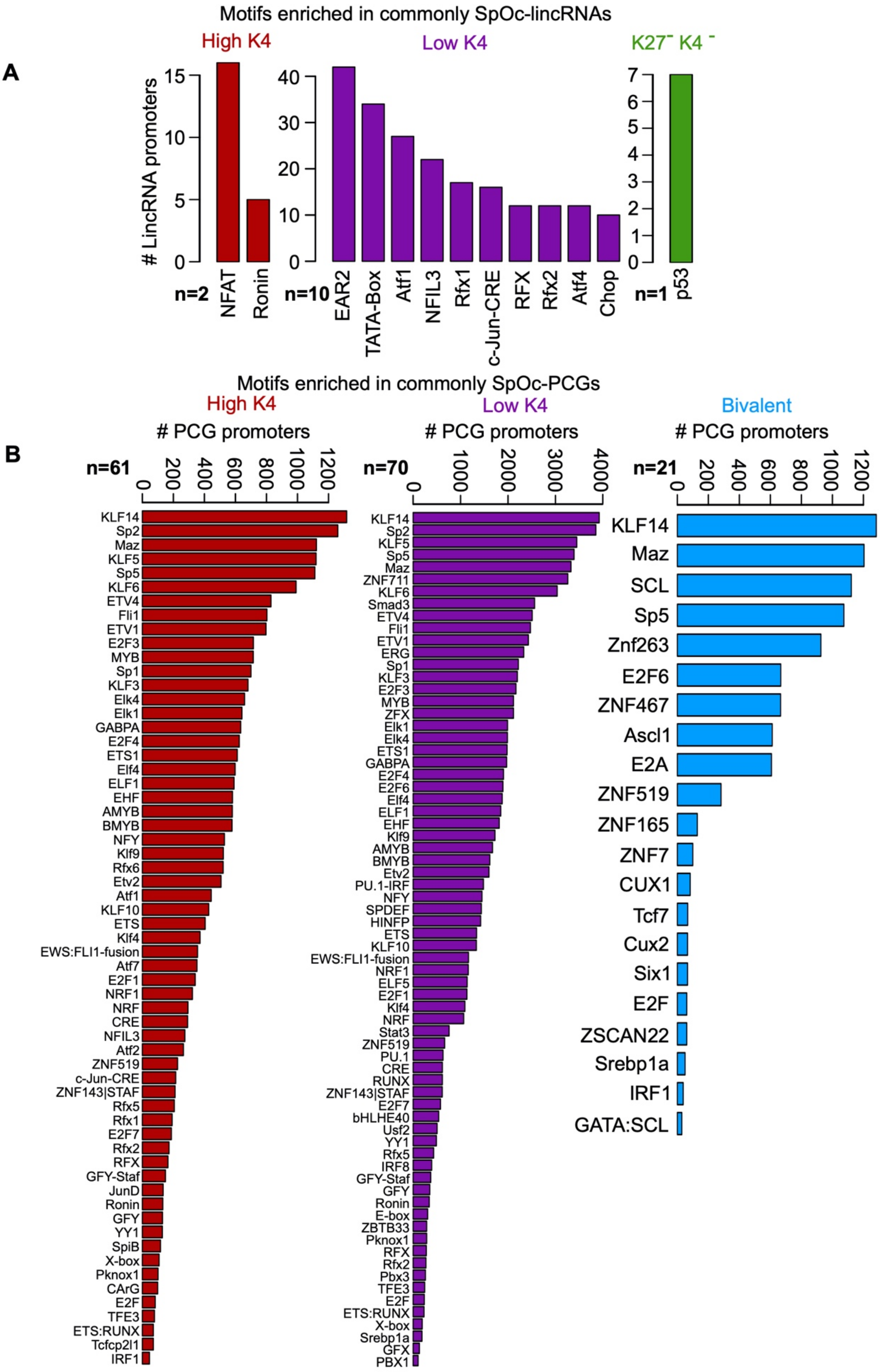

Figure S5. Frequency of transcription factor motifs in human SpOc expressed transcripts, Related to Figure 7. A-B) Bar graphs showing the number of associated human SpOc-lincRNA (**A**) and SpOc-PCG (**B**) promoters from three sperm derived chromatin clusters of SpOc transcripts enriched with the indicated transcription factor motifs.

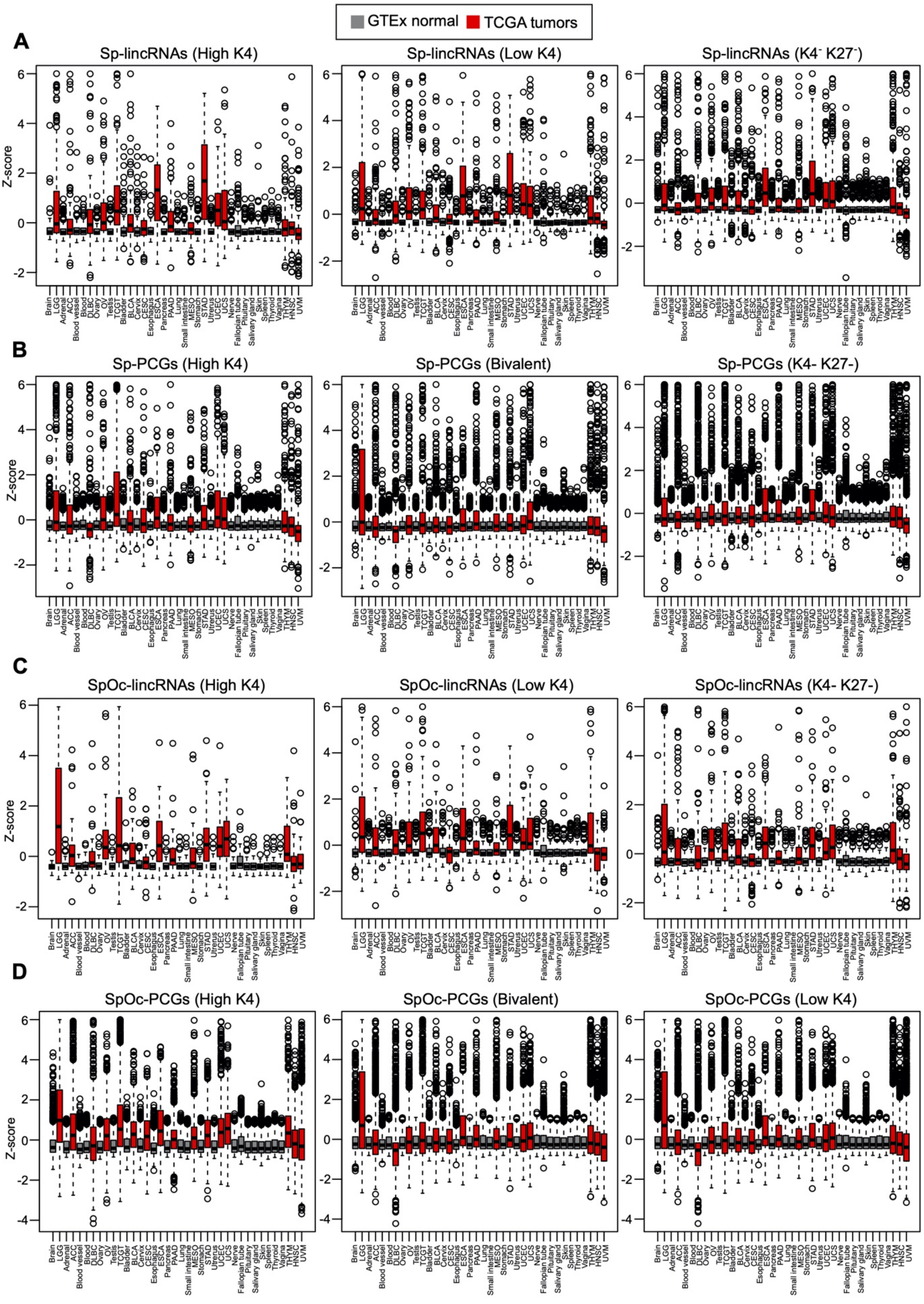

Figure S6. Aberrant expression of Sp and SpOc transcripts in different cancers, Related to Figure 8. A-D) Expression status of sperm derived chromatin clusters of Sp-transcripts (Friedman et al.) and SpOc-transcripts (**C-D**) in TCGA tumor patient cohorts and healthy samples from GTEx. The z-score in the plots were derived from the normalized TPM values.

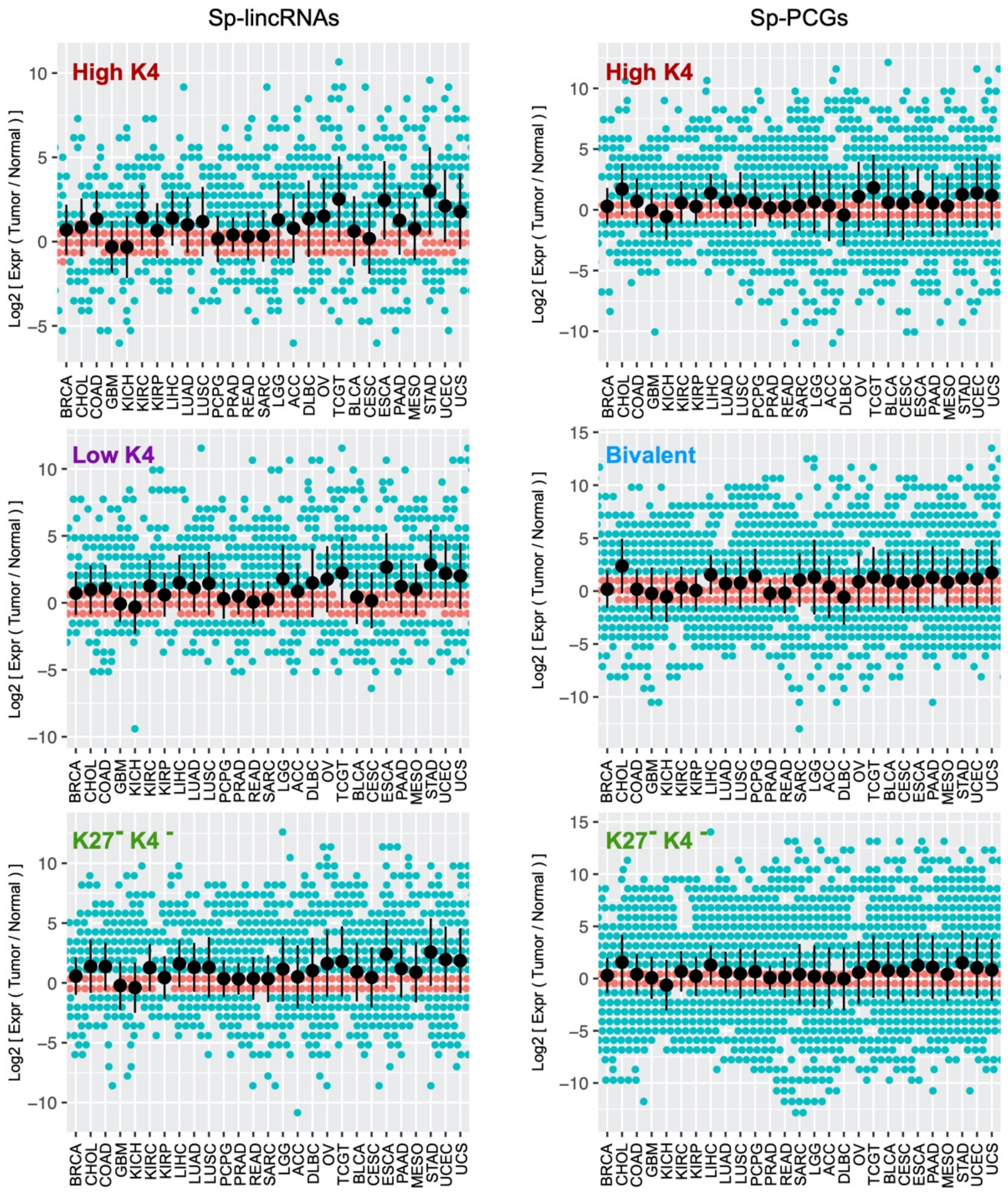

Figure S7. Directional regulation status of Sp transcripts in different cancers, Related to Figure 8. A-B) Fold differences of Sp-lincRNAs (**A**) and Sp-PCGs (**B**) from sperm derived chromatin clusters by comparing TCGA tumors with normal healthy tissues. The fold changes are calculated from normalized TPM values.

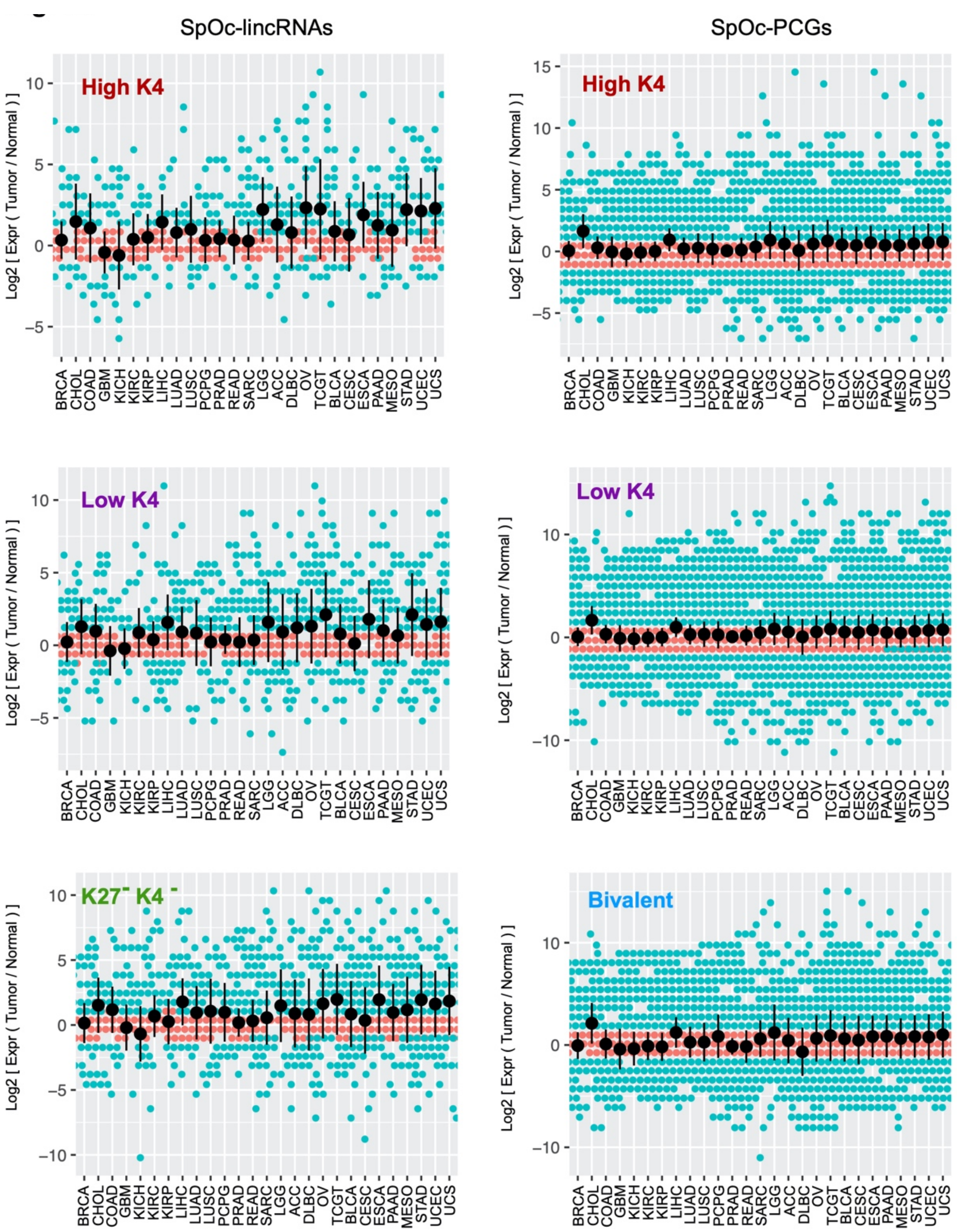

Figure S8. Directional regulation status of Sp transcripts in different cancers, Related to Figure 8. A-B) Fold differences of SpOc-lincRNAs (**A**) and SpOc-PCGs (**B**) from sperm derived chromatin clusters by comparing TCGA tumors with normal healthy tissues. The fold changes are calculated from normalized TPM values.

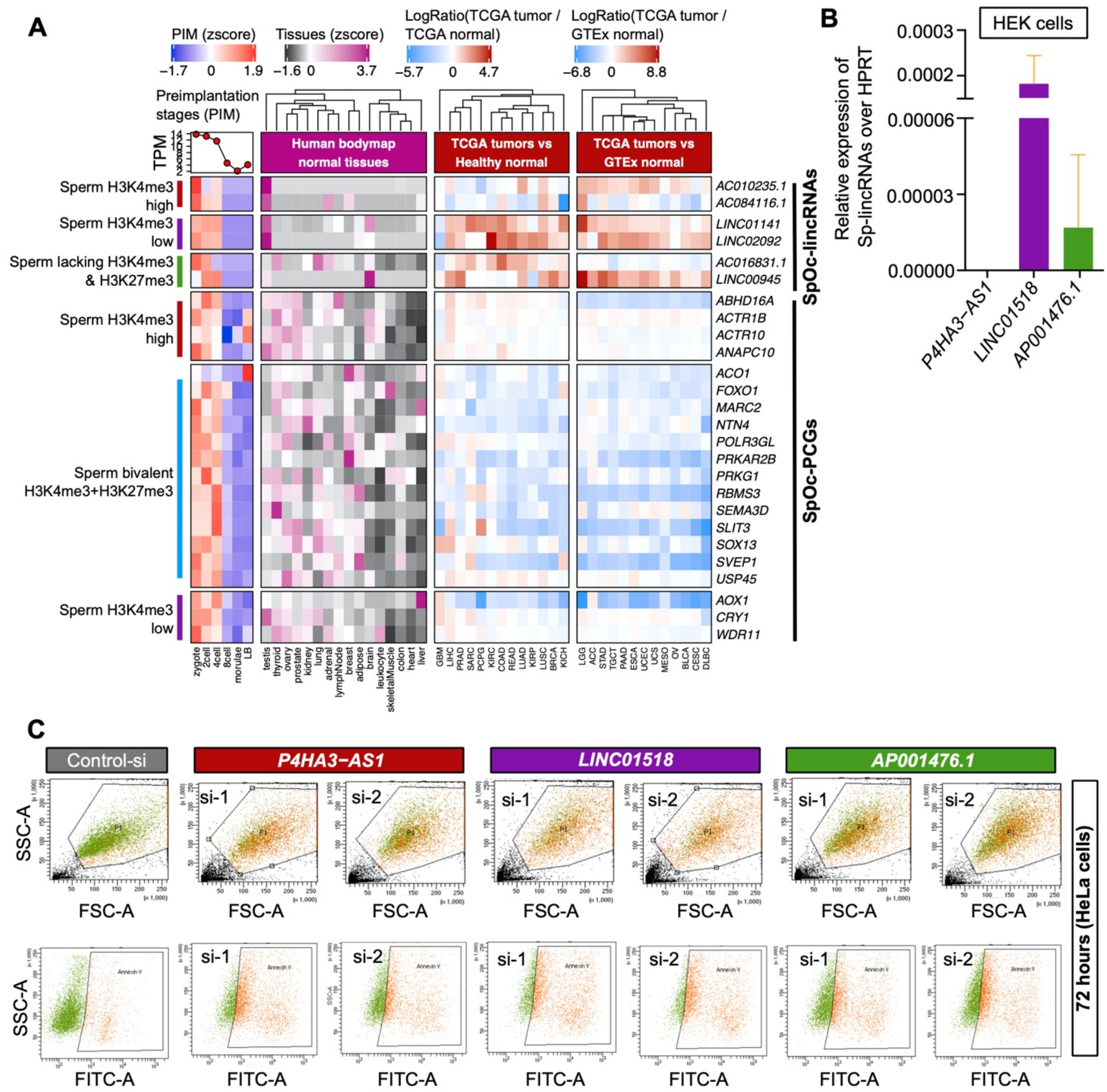

Figure S9. SpOc transcripts show tumor-specific activation, Related to Figure 9. A) Status of SpOc transcripts in germ cells, preimplantation stage embryos, the human body map tissues, TCGA tumors compared with the corresponding healthy samples or TCGA tumors compared with GTEx health samples. Z-score in the plots is derived from the normalized TPM expression values. The log fold change is calculated by comparing the expression of tumors with the health samples expression. **B**) Relative expression of Sp transcripts in HEK cell line. Data from barplot is represented as mean ± SD. **C**) Scatter plots showing the Hela cells stained with Alexa Fluor 488 annexin V conjugate, which is green Fluorescein (FITC) dye after 72hrs of transfection with siRNA for Sp lincRNAs and control siRNA samples. Single cells (green dots) were gated from debris (black dots) using a forward scatter area (FSC-A) versus side scatter area (SSC-A) gate (P1). Red colored dots represent Annexin V positive staining cells. Two different siRNAs were used for each gene (si1 and si2).

Table S4. siRNA sequence used in this study, Related to Figure 9 and Figure S9.

Oligo Name	Sequence (5' - 3')
AP001476.1_si1	CGAAGUCUCCUUGUCAAGA
AP001476.1_si2	CCGCUAUUUGGGCGGUGUU
P4HA3-AS1_si1	CCACACAAUAGUGAGAUAU
P4HA3-AS1_si2	GUGAGAUGUUAGGCCAUUA
LINC01518_si1	GGGAUCAUGGCCAAAGUUG
LINC01518_si2	GGCAGUGACGGAACAGUAC

Transparent Methods

Data collection

We collected 17,705 RNA-seq and ChIP-seq datasets from different studies, GEO, ENCODE, dbGAP, GTEx and TCGA. The detailed list of samples and their sources are provided in **Table S1**. Datasets are chosen after quality check using FastQC (RNA-seq and ChIP-seq), SAMstat(Lassmann et al., 2011) (RNA-seq and ChIP-seq) and deepTools(Ramirez et al., 2016) fingerprints (ChIP-seq vs input). Transcriptome data for different cancers are downloaded as raw read counts using Bioconductor package TCGAbiolinks(Colaprico et al., 2016; Mounir et al., 2019; Silva et al., 2016) and the raw read count data for tissues used for comparison with TCGA patients are downloaded from GTEx consortium.

Processing transcriptome dataset (RNA-seq)

All downloaded RNA-seq samples were first subjected to quality check with FastQC before and after adaptor removal using cutadapt (if necessary). Samples were considered for further analysis only when it passes FastQC quality check. We used HISAT2 (v2.1.0)(Kim et al., 2019) to align reads to hg38 (human) or mm10 (mouse) with parameters *'--dta --avoid-pseudogene --known-splicesite- --qc-filter'* and *'--rna-strandness'* if the sequencing samples are paired-end. The known splice sites are generated from Ensembl annotation(Yates et al., 2019) GRCh38.90 (human) or GRCm38.90 (mouse) using 'hisat2_extract_splice_sites.py' and the genomes are indexed using 'hisat2-build'. The generated aligned SAM files are sorted and converted into BAM files with the help of SAMtools (v 1.5)(Li et al., 2009). Alignment quality and statistics are obtained using SAMstat. For assigning reads to individual transcripts (genes or RNAs) from Ensembl annotation GRCh38.90 (human) or GRCm38.90 (mouse), featureCounts(Liao et al., 2014) from Subread Bioconductor package was employed using parameters *'-Q 30 -t exon --ignoreDup -J –minOverlap -C'* and *'-B -s'* for paired-end and strand-specific samples. Obtained raw read counts for individual transcripts are normalized as Transcripts Per Kilobase Million (TPM) by using library size and length of exons. If necessary, batch effects were removed from normalized reads using 'removeBatchEffect' from Bioconductor package.
According to Hammoud SS et al 2014, both the sperm donors were similar in age (D1-32 and D2-37), both white-Caucasian, comparable semen parameters (progressive motility ~65%, sperm count ~$80x10^6$). Oocytes samples were obtained from Yan L et al 2013 in which the couples who had more than 20 oocytes derived from the same IVF cycle were sequenced. Embryos that were produced by routine fertilization were cultured individually. The women had an average age of 30 years (25–35 years). Detailed information on the studies associated with entire dataset used in the current analysis can be found in **Table S1**.

Processing chromatin immunoprecipitation data (ChIP-seq)

After FastQC quality check, raw reads from ChIP-seq were aligned using BWA (v 0.7.17) aligner(Li and Durbin, 2009) and obtained SAM files are aligned and converted into BAM using SAMtools (v 1.5). De-duplication of reads was done using Picard MarkDuplicates (v 2.18.11)(McKenna et al., 2010) and the known blacklisted regions are removed using BEDTools '*intersect*'(Quinlan and Hall, 2010). Additionally, we removed low quality reads having mapping quality less than 30 using *bamutils* from NGSUtils(Breese and Liu, 2013). Finally, the BAM files with no duplicate reads and higher mapping quality were used for downstream analysis. To check quality of enrichment in ChIP-seq, we compared read coverage of ChIP-seq against the input/control samples using

‘plotFingerprint’ from deepTools package (v 2.5.1). Only samples with higher enrichment were used in bamCompare (deepTools) to obtain base-by-base resolution of genome-wide fold enrichment (log2ratio/enrichment) by comparing ChIP-seq with input samples *(--scaleFactorsMethod readCount --ratio log2 --pseudocount 1*). These base-by-base enrichment values are plotted for promoters of genes using’ plotHeatmap’ from deepTools. Promoters of Sp and SpOc transcripts are clustered by chromatin profiles using k-means clustering and tested up to 10 clusters to find optimal number of clusters. We optimized the cluster numbers by considering only non-repetitive combination (H3K27me3 and H3K4me3) of clusters. The protein coding genes (PCGs) from individual clusters are used for further functional enrichment analysis. We used GeneSCF(Subhash and Kanduri, 2016) to derive enriched biological process from individual clusters by using gene ontology from human and mouse. Detailed information on the studies associated with entire dataset used in the current analysis can be found in **Table S1**.

Motif analysis of Sp transcript promoters

Promoter sequences of Sp and SpOc transcripts are extracted by considering ± 250 base-pairs (bp) from transcription start site (TSS). The FASTA sequences of these promoters were extracted using ‘*faidx*’ from SAMtools (v 1.5). To find transcription factor motif sequences or binding sites on this promoter, we used ‘*findMotifs.pl*’ (*-mset vertebrates -homer2*) from HOMER (v 4.10) package(Duttke et al., 2019). HOMER matches provided sequences with known transcription factor motifs predicted from published ChIP-seq datasets and ranks by its significance. We considered motif to be enriched only if it has p-value < 0.01. Also, top enriched motifs from Sp and SpOc transcript promoters are selected by highest number of target genes having particular motif binding site.

TCGA data access and processing

The raw read counts for TCGA patient data and its corresponding controls were downloaded using TCGAbiolinks. Obtained raw reads are normalized to TPM and calculated average expression of patients and normal sample groups per gene.

RNA extraction, cDNA synthesis and qRT-PCR

Total RNA was isolated from cells using Relia RNA isolationkit (Promega) following manufacturer’s directions. cDNA synthesis was carried out using ImProm-II Reverse Transcriptase (Promega). RT-qPCR analysis was done using Power SYBR Green PCR master mix (Applied Biosystems, Warrington, UK). Differences in expression were calculated using the ΔΔCt method using HPRT as control gene. The primers used for qRT-PCR are 5’AAGAGCGGCAAGAGGACAG3’ and 5’ GGACTATGCAGTTCCTTCCTG3’ for *P4HA3-AS1*; 5’ ACTGAAGCTGGTGGCTGTG3’ and 5’ CATGGACTCGAGAGCTGACA3’ for *LINC01518* and 5’ ACTGCCACTCGGACTGTCTC3’ and 5’ GAAAGGCACTCTGTGGTCGT3’ for *AP001476.1*. Two Custom designed small interfering RNAs (siRNAs) for each lncRNA were used for transfections along with control siRNA (Invitrogen).

MTT assay and Cell cycle analysis

Percentage of cell proliferation was analyzed using MTT assay after 48 h of post transfection according to the manufacturer’s protocol using CellTiter-Glo® 3D Cell Viability Assay (G9681, Promega Madison, USA). Proliferation capacity of HeLa cells as measured based on the Ultra-Glo Recombinant Luciferase, which generates a stable luminescent signal measured using Clariostar Plus Microplate Reader (BMG labtech). The error bars were calculated based on two independent transfections. The cell cycle profiling was performed using NucleoCounter NC-3000 platform (Chemometec, Denmark). The cells were fixed using absolute ethanol after transfection and were stained with DAPI solution provided by the manufacturer and analyzed according to manufacturer’s instructions. All siRNA sequences used in this study are listed in **Table S4**.

Apoptosis assay

Hela cells are stained with Alexa Fluor 488 annexin V conjugate, which is green Fluorescein (FITC) dye after 48hrs and 72hrs of transfection with siRNA for Sp-lincRNAs and control siRNA samples. Single cells were gated from debris using a forward scatter area (FSC-A) versus side scatter area (SSC-A) gate (P1). We measured Annexin V positively stained cells in cells transfected with two different siRNAs for each Sp-lincRNAs (si1 and si2) along with corresponding control siRNAs. All siRNA sequences used in this study are listed in **Table S4**.

Supplemental References

1. Breese, M.R., and Liu, Y. (2013). NGSUtils: a software suite for analyzing and manipulating next-generation sequencing datasets. Bioinformatics *29*, 494-496.
2. Colaprico, A., Silva, T.C., Olsen, C., Garofano, L., Cava, C., Garolini, D., Sabedot, T.S., Malta, T.M., Pagnotta, S.M., Castiglioni, I.*, et al.* (2016). TCGAbiolinks: an R/Bioconductor package for integrative analysis of TCGA data. Nucleic Acids Res *44*, e71.
3. Duttke, S.H., Chang, M.W., Heinz, S., and Benner, C. (2019). Identification and dynamic quantification of regulatory elements using total RNA. Genome Res *29*, 1836-1846.
4. Friedman, C.E., Nguyen, Q., Lukowski, S.W., Helfer, A., Chiu, H.S., Miklas, J., Levy, S., Suo, S., Han, J.J., Osteil, P.*, et al.* (2018). Single-Cell Transcriptomic Analysis of Cardiac Differentiation from Human PSCs Reveals HOPX-Dependent Cardiomyocyte Maturation. Cell Stem Cell *23*, 586-598 e588.
5. Kim, D., Paggi, J.M., Park, C., Bennett, C., and Salzberg, S.L. (2019). Graph-based genome alignment and genotyping with HISAT2 and HISAT-genotype. Nat Biotechnol *37*, 907-915.
6. Lassmann, T., Hayashizaki, Y., and Daub, C.O. (2011). SAMStat: monitoring biases in next generation sequencing data. Bioinformatics *27*, 130-131.
7. Li, H., and Durbin, R. (2009). Fast and accurate short read alignment with Burrows-Wheeler transform. Bioinformatics *25*, 1754-1760.
8. Li, H., Handsaker, B., Wysoker, A., Fennell, T., Ruan, J., Homer, N., Marth, G., Abecasis, G., Durbin, R., and Genome Project Data Processing, S. (2009). The Sequence Alignment/Map format and SAMtools. Bioinformatics *25*, 2078-2079.
9. Liao, Y., Smyth, G.K., and Shi, W. (2014). featureCounts: an efficient general purpose program for assigning sequence reads to genomic features. Bioinformatics *30*, 923-930.
10. McKenna, A., Hanna, M., Banks, E., Sivachenko, A., Cibulskis, K., Kernytsky, A., Garimella, K., Altshuler, D., Gabriel, S., Daly, M.*, et al.* (2010). The Genome Analysis Toolkit: a MapReduce framework for analyzing next-generation DNA sequencing data. Genome Res *20*, 1297-1303.
11. Mounir, M., Lucchetta, M., Silva, T.C., Olsen, C., Bontempi, G., Chen, X., Noushmehr, H., Colaprico, A., and Papaleo, E. (2019). New functionalities in the TCGAbiolinks package for the study and integration of cancer data from GDC and GTEx. PLoS Comput Biol *15*, e1006701.
12. Quinlan, A.R., and Hall, I.M. (2010). BEDTools: a flexible suite of utilities for comparing genomic features. Bioinformatics *26*, 841-842.
13. Ramirez, F., Ryan, D.P., Gruning, B., Bhardwaj, V., Kilpert, F., Richter, A.S., Heyne, S., Dundar, F., and Manke, T. (2016). deepTools2: a next generation web server for deep-sequencing data analysis. Nucleic Acids Res *44*, W160-165.
14. Silva, T.C., Colaprico, A., Olsen, C., D'Angelo, F., Bontempi, G., Ceccarelli, M., and Noushmehr, H. (2016). TCGA Workflow: Analyze cancer genomics and epigenomics data using Bioconductor packages. F1000Res *5*, 1542.
15. Subhash, S., and Kanduri, C. (2016). GeneSCF: a real-time based functional enrichment tool with support for multiple organisms. BMC Bioinformatics *17*, 365.
16. Yates, A.D., Achuthan, P., Akanni, W., Allen, J., Allen, J., Alvarez-Jarreta, J., Amode, M.R., Armean, I.M., Azov, A.G., Bennett, R.*, et al.* (2019). Ensembl 2020. Nucleic Acids Res.
